# Single-cell sequencing of ascites fluid illustrates heterogeneity and therapy-induced evolution during gastric cancer peritoneal metastasis

**DOI:** 10.1038/s41467-023-36310-9

**Published:** 2023-02-14

**Authors:** Xuan-Zhang Huang, Min-Jiao Pang, Jia-Yi Li, Han-Yu Chen, Jing-Xu Sun, Yong-Xi Song, Hong-Jie Ni, Shi-Yu Ye, Shi Bai, Teng-Hui Li, Xin-Yu Wang, Jing-Yuan Lu, Jin-Jia Yang, Xun Sun, Jason C. Mills, Zhi-Feng Miao, Zhen-Ning Wang

**Affiliations:** 1 Department of Surgical Oncology and General Surgery, The First Hospital of China Medical University, 155 N Nanjing Street, Shenyang, Liaoning China; 2 Key Laboratory of Precision Diagnosis and Treatment of Gastrointestinal Tumors, Ministry of Education, China Medical University, Shenyang, Liaoning China; 3 Institute of Health Sciences, China Medical University, Shenyang, Liaoning China; 4 Eight-year system, Institute of innovation, China Medical University, Shenyang, Liaoning province, Shenyang, Liaoning China; 5 Department of Immunology, China Medical University, Shenyang, Liaoning China; 6 Section of Gastroenterology & Hepatology, Department of Medicine, Baylor College of Medicine, 535E Anderson-Jones Building, One Baylor Plaza, Houston, TX USA; 7 Department of Pathology & Immunology, Baylor College of Medicine, 535E Anderson-Jones Building, One Baylor Plaza, Houston, TX USA; 8 Department of Molecular and Cellular Biology, Baylor College of Medicine, 535E Anderson-Jones Building, One Baylor Plaza, Houston, TX USA

**Keywords:** Gastric cancer, Cancer microenvironment, Cancer microenvironment

## Abstract

Peritoneal metastasis is the leading cause of death for gastrointestinal cancers. The native and therapy-induced ascites ecosystems are not fully understood. Here, we characterize single-cell transcriptomes of 191,987 ascites cancer/immune cells from 35 patients with/without gastric cancer peritoneal metastasis (GCPM). During GCPM progression, an increase is seen of monocyte-like dendritic cells (DCs) that are pro-angiogenic with reduced antigen-presenting capacity and correlate with poor gastric cancer (GC) prognosis. We also describe the evolution of monocyte-like DCs and regulatory and proliferative T cells following therapy. Moreover, we track GC evolution, identifying high-plasticity GC clusters that exhibit a propensity to shift to a high-proliferative phenotype. Transitions occur via the recently described, autophagy-dependent plasticity program, paligenosis. Two autophagy-related genes (*MARCKS* and *TXNIP*) mark high-plasticity GC with poorer prognosis, and autophagy inhibitors induce apoptosis in patient-derived organoids. Our findings provide insights into the developmental trajectories of cancer/immune cells underlying GCPM progression and therapy resistance.

## Abstract (web summary)

Peritoneal metastasis is one of the most common forms of death for gastrointestinal cancers, however, its cell composition is incompletely understood. Here, the authors use single cell RNA-seq of peritoneal metastases from 35 patients and show diversity in immune cells, and plasticity in cancer cell phenotypes and autophagy related genes as biomarkers of prognosis.

## Introduction

Gastric cancer (GC) is the fifth most-diagnosed cancer and the fourth leading cause of cancer deaths worldwide^1^. Gastric cancer peritoneal metastasis (GCPM) is common after curative surgical resection and portends a poor prognosis of <6 months overall survival^2–4^. GC patients diagnosed with GCPM during the perioperative stage experience only limited benefits from anti-tumor therapy strategies^5–8^. The molecular mechanisms of GCPM occurrence and development remain poorly understood. Therefore, an in-depth and dynamic exploration of GCPM occurrence and development can help us elucidate mechanisms involved in GCPM molecular pathology and find more effective therapeutic targets for GCPM.

GCPM occurs when GC cells selectively find a suitable ecosystem for growth in the peritoneum^9–11^. The peritoneal ecosystem is highly complex, including distinct heterogeneous immune cell populations^12,13^. Both peritoneal exfoliated tumor cells (PETCs) and immune cells in the abdominal cavity undergo diverse changes during GCPM development, and anti-tumor therapy might lead to a therapy-induced evolution of GCPM cells, in turn affecting their sensitivity to therapy^14–16^. Comprehensive dissection of the characteristics of peritoneal infiltrating immune cells and PETCs with single-cell resolution is necessary to better understand the underlying molecular mechanisms of GCPM and provide insights into future therapy strategies. Several recent studies dissected intratumoral heterogeneity and lineage diversity in primary GC and peritoneal metastatic foci^17,18^. However, the dynamic heterogeneity of the peritoneal ecosystem in early GCPM, the most promising clinical intervention stage, and the therapy-induced evolution of cells within this ecosystem, especially immune checkpoint evolution, remain enigmatic.

In this study, we characterized a total of 191,987 high-quality cells from 35 patients across five groups relative to GCPM development and treatment status. We assessed the diversity and heterogeneity of cells in the peritoneal ecosystem and observed proportionally reduced dendritic cells (DCs) and increased regulatory CD4 T cells (Treg) and naïve T cells during GCPM evolution. Notably, monocyte-like DCs exhibited high diversity and pro-angiogenic phenotypes, with reduced antigen-presenting capacity and increased pro-angiogenic capacity. We describe a proliferative cycling T cell cluster in the peritoneal ecosystem which represents an exhausted and dysfunctional stage of T cells comprising three heterogeneous sub-clusters. Peritoneal infiltrating monocyte-like DCs and cycling T cells exhibited marked evolution of heterogeneity after therapy, involving cell fate transition, immune phenotype changes, and metabolic reprograming. For GC cells, high-plasticity GC cells were marked by a propensity to evolve after therapy into high-proliferative GC cells. The plasticity transition from quiescent to proliferative states resembled the recently described plasticity process called paligenosis. Paligenosis was originally defined as the program by which differentiated (mitotically quiescent) cells use autophagy and dynamic mTORC1 regulation to reprogram into dividing cells in precancerous lesions like metaplasia^19^. This process has been proposed that if GC arises from metaplasia that arose via paligenosis, then perhaps GC cells may use paligenosis to survive^20^. Accordingly, we show here that two autophagy-related genes (*MARCKS* and *TXNIP*) were identified as biomarkers of high-plasticity GC. Furthermore, autophagy and mTORC1 inhibitors (known to block paligenosis) significantly induced apoptosis in patient-derived organoids (PDOs) from ascites samples. Our large-scale single-cell dataset provides a direction for future research on the molecular mechanisms of GCPM and will help design effective therapy strategies for GCPM in the future.

## Results

### Dynamic changes in the cellular ecosystem in ascites or peritoneal lavage fluid from GC patients

**Fig. 1:**
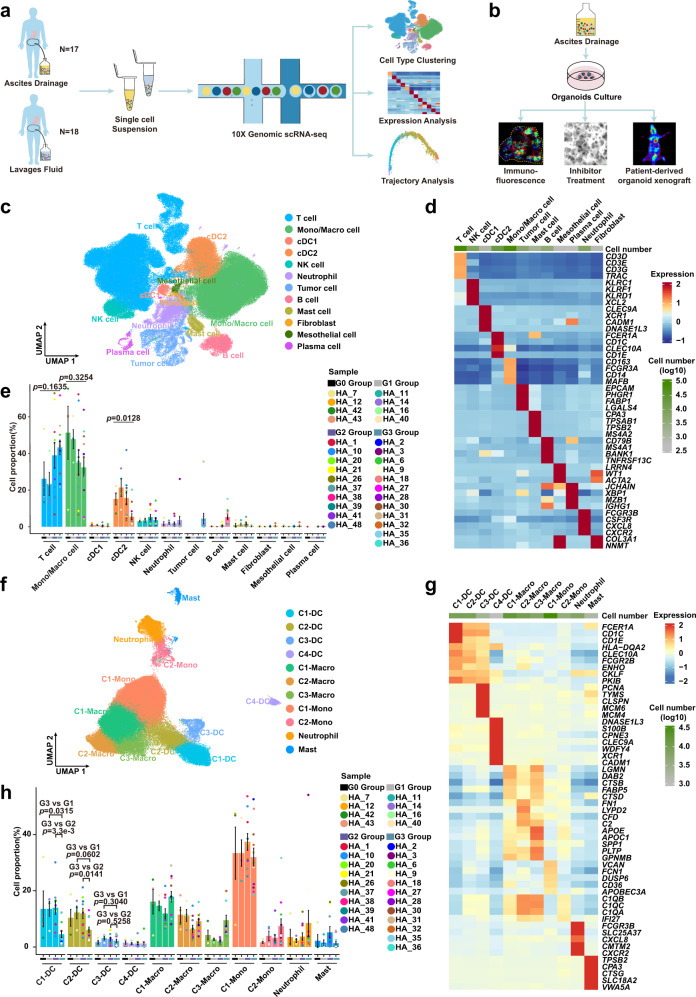
scRNA-seq profiles of dynamic changes in the peritoneal ecosystem. **a** Scheme of the experimental design and analytical workflow of this study for scRNA-seq. **b** Validation experiment based on patient-derived organoids from ascites. **c** Uniform Manifold Approximation and Projection (UMAP) plot showing the main cell types from all samples. Each cluster is colored and annotated according to cell type. Mono/Macro, monocyte/macrophage; cDC1, type 1 conventional dendritic cells; cDC2, type 2 conventional dendritic cells. **d** Heatmap showing z-score normalized mean expression of selected marker genes in each cell type. **e** The proportion of each cell type in different groups from G0 (*n* = 4 samples), G1 (*n* = 4 samples), G2 (*n* = 10 samples) and G3 (*n* = 12 samples) Group. Histogram colors correspond to cell type colors in **c**; point colors correspond to samples. Data are presented as mean values ± SEM (error bars); the *p*-values are calculated by one-way ANOVA test. **f** UMAP plot representing myeloid cell clusters colored by cluster. DC dendritic cell, Macro macrophage, Mono monocyte. **g** Heatmap showing z-score normalized mean expression of selected genes in each cluster of myeloid cells. **h** The proportion of each cluster of myeloid cells from G0 (*n* = 4 samples), G1 (*n* = 4 samples), G2 (*n* = 10 samples) and G3 (*n* = 12 samples) Groups. Histogram colors correspond to cell type colors in **f**; point colors correspond to samples. Data are presented as mean values ± SEM (error bars); the *p-*values are calculated by two-sided unpaired Student’s *t*-test. Source data are provided as a Source Data file.

We obtained scRNA-seq transcriptomic profiles of cells from the peritoneal ecosystem from 35 patients across four medical centers, including normal peritoneal lavage fluid from four benign hysteromyoma patients (normal negative controls, G0 Group), peritoneal lavage fluid from four early GC patients (G1 Group); peritoneal lavage fluid from 10 advanced GC patients (G2 Group); ascites from 12 untreated advanced GC patients with diagnosed GCPM (G3 Group); and ascites from five advanced GC patients with diagnosed GCPM following systemic therapy (G4 Group) (Fig. 1a and Supplementary Table 1). We additionally collected four ascites samples from untreated advanced GC patients with diagnosed GCPM to culture ascites PDOs for experimental validation based on inhibitor drug intervention experiments and immunofluorescence assays (Fig. 1b and Methods).

To ensure that the filtered scRNA-seq data for further downstream analysis were from single live cells, we used the harmony algorithm to integrate scRNA-seq data from samples from the five groups and applied strict quality control and cell filtering (Supplementary Table 2). A total of 191,987 high-quality cells including myeloid cells, lymphocytes, fibroblast cells, mesothelial cells, and tumor cells were ultimately obtained (Fig. 1c), with an average of 2,744 genes, 11,177 unique molecular identifiers (UMIs), and only about 6% mitochondrial genes per cell detected (Supplementary Fig. 1a, b and Supplementary Table 2). We identified 12 major cell clusters including immune and non-immune cells using the Uniform Manifold Approximation and Projection (UMAP) method (Fig. 1c). All cells had high expression levels of housekeeping genes such as *GAPDH*, *ACTB*, *B2M*, and *RPL11* (Supplementary Fig. 1c), verifying the quality and accuracy of single cells. Immune and non-immune cells were divided based on *PTPRC* expression (Supplementary Fig. 1c).

Immune cells accounted for the majority of our analyzed cells from the peritoneal ecosystem, consistent with previous findings from peritoneal dialysis fluid^13^. We identified multiple immune cell types using the following markers: T cells (*CD3D* and *CD3E*), NK cells (*KLRC1* and *KLRF1*), type 1 conventional DCs (cDC1, *CLEC9A* and *XCR1*), type 2 conventional DCs (cDC2, *CLEC10A* and *CD1C*), macrophages/monocytes (*CD163*, *FCGR3A* and *CD14*), mast cells (*CPA3* and *TPSB2*), B cells (*CD79B* and *MS4A1*), plasma cells (*XBP1* and *MZB1*), and neutrophils (*FCGR3B* and *CSF3R*) (Fig. 1c, d and Supplementary Fig. 1c). For non-immune cell populations, we identified tumor cells (*EPCAM* and *CLDN4*), fibroblasts (*COL3A1* and *NNMT*), and mesothelial cells (*LRRN4* and *WT1*) (Fig. 1c, d and Supplementary Fig. 1c). Inferring chromosomal copy number variations (inferCNV) including gain and loss of chromosomes based on transcriptome has been widely used to identify whether epithelial cells are malignant tumor cells across scRNA-seq studies^21–24^. Our results showed that all identified epithelial cells were confirmed as malignant tumor cells by inferCNV analysis (Supplementary Fig. 1e, f). Over twelve cell clusters exhibited distinct distributions during the GCPM progression, with macrophages/monocytes and T cells, which were the main cell composition in the peritoneal cavity, exhibiting decreasing and increasing trends, respectively (Fig. 1e and Supplementary Fig. 1d). We next conducted ELISA assays to measure the concentrations of chemokines in the peritoneal cavity to explore their effects on recruitment of immune cells into the peritoneal cavity during GCPM progression. CXCL16, a chemokine which recruits T cells, was elevated during GCPM (*p* = 5.9e-5), consistent with the change in the proportion of T cells, while the concentrations of chemokines that recruit macrophages/monocytes displayed complex changes (Supplementary Fig. 1g)^25–27^. The concentration of CCL5 (*p* = 0.0004) was reduced, but CCL2 (*p* = 1.6e-5), CCL3 (*p* = 0.0125) and CCL4 (*p* = 0.0051) were increased during GCPM progression (Supplementary Fig. 1g). The inconsistent changes of the macrophages/monocytes proportions and monocyte-recruitment chemokines may be due to the fact that monocyte-recruitment chemokines can also simultaneously recruit other cells, such as T cells, NK cells, neutrophils, DCs and MDSCs, making the recruitment of monocytes simultaneously influenced by multiple cytokines^25–27^. The concentrations of chemokines found via ELISA were consistent with the changes of their transcripts in our scRNA-seq data (Supplementary Fig. 1h).

### Monocyte-like dendritic cells exhibit high diversity and a pro-angiogenic phenotype during GCPM

We first analyzed the G0-G3 Group to explore the immune cell landscape and the dynamic changes in immune cells during GCPM. Myeloid cells dominate the immune cell population. The cDC2 cluster displayed obvious variation among different groups, and the proportion of these cells infiltrating into the peritoneal cavity decreased as GC progressed, specifically in patients with GCPM (*p* = 0.0128) (Fig. 1e). Macrophages/monocytes showed a decreasing trend without statistical significance (*p* = 0.3254) (Fig. 1e). To explore potential distinct functional roles of cell clusters, we performed further myeloid cell clustering. In total, 11 cell clusters were identified among myeloid lineages: four DC clusters, three macrophage clusters, two monocyte clusters, one neutrophil cluster, and one mast cell cluster (Fig. 1f and Supplementary Fig. 1i).

**Fig. 2:**
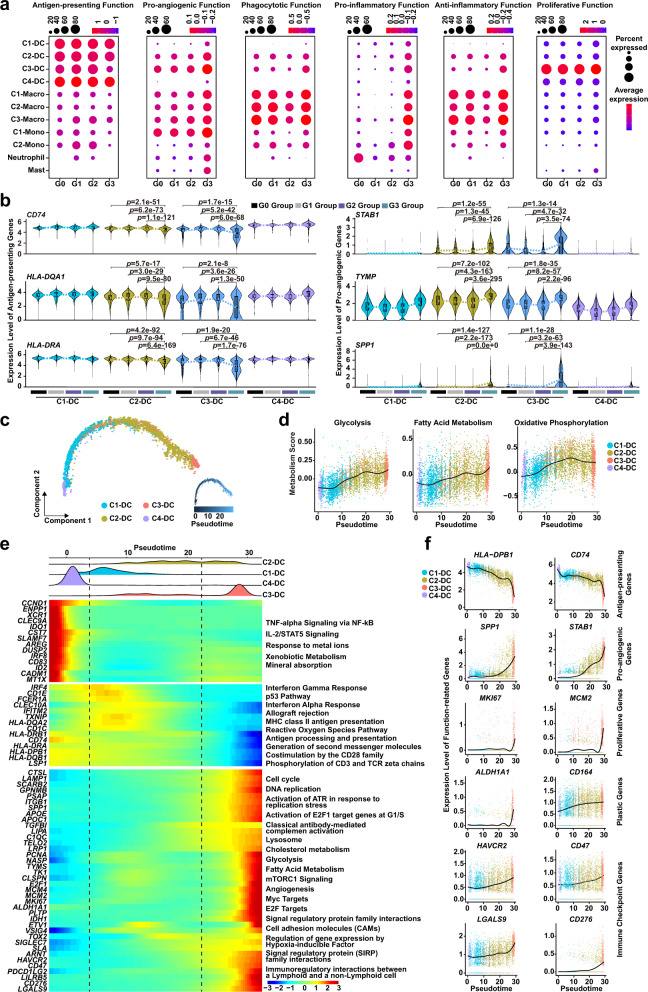
Monocyte-like dendritic cells exhibit high diversity and a pro-angiogenic phenotype during GCPM. **a** Dotplot showing antigen-presenting, pro-angiogenic, phagocytotic, pro-inflammatory, anti-inflammatory, and proliferative function scores of myeloid-derived cell clusters in G0-G3 Groups. Dot size represents percent of expressing cells in each cluster, color represents z-score of normalized mean expression level of selected gene signatures. DC dendritic cell, Macro macrophage, Mono monocyte. **b** Violin plot showing expression levels of selected antigen-presenting and pro-angiogenic genes in dendritic cell (DC) clusters among G0 (*n* = 4 samples), G1 (*n* = 4 samples), G2 (*n* = 10 samples) and G3 (*n* = 12 samples) Group, color-coded by cell type. Horizontal dotted line represents mean value, and colored dotted curve line indicates changes in expression level of selected genes. Box represents median ± interquartile range; whiskers represent 1.5x interquartile range; *p*-values are calculated by two-sided unpaired Wilcoxon test. **c** Developmental trajectory plot of dendritic cell (DC) clusters, color-coded by cluster (left) and pseudotime (right). Each dot represents a single cell. **d** Curve plots showing metabolism score changes related to glycolysis, fatty acid metabolism, and oxidative phosphorylation in dendritic cells (DCs) along pseudotime. Point colors correspond to cell type colors in **c**. **e** The cell distribution of each dendritic cell (DC) cluster along with the pseudotime (upper panel), color-coded by DC clusters. Heatmap showing dynamic expression changes of genes in DC clusters (lower panel). **f** Curve plots showing expression level changes of function-related genes related to antigen-presentation, pro-angiogenesis, proliferation, plasticity, and immune checkpoint along pseudotime in dendritic cells (DCs). Point colors correspond to cell type colors in **c**. Source data are provided as a Source Data file.

DCs are the main regulators for initiating antigen-specific immune responses in tumor immunity. We observed distinct *CLEC9A*^*+*^*/XCR1*^*+*^ cDC1 and *CLEC10A*^*+*^*/CD1C*^*+*^ cDC2 clusters (Fig. 1g)^28–30^. Our C4-DC cluster, featuring high expression of *CLEC9A* and *XCR1*, was identified as cDC1, with a relatively low proportion (Fig. 1f,g,h). cDC2 cells were divided into three clusters (C1-DC, C2-DC and C3-DC) (Fig. 1f,g and Supplementary Fig. 2a), characterized by comparable expression of major histocompatibility complex (MHC)-II and lower expression of MHC-I, consistent with previous reports of cDC1s and cDC2s mainly initiating CD8 and CD4 T cells responses, respectively (Supplementary Fig. 2a)^28,31,32^. C2-DC and C3-DC clusters highly expressed *CD14, CD163, FCN1, MAFB, S100A9*, and *FCGR1A*, while *CD1C* and *MHC-II* levels were relatively lower; this gene expression profile classified those clusters as more monocytic (monocyte-like DCs)^33,34^ (Supplementary Fig. 2a, b). C3-DC is a cDC2 cluster characterized by high *MCM4, MCM6*, and *PCNA* expression, with lower antigen-presenting capacity and higher pro-angiogenic capacity (characterized by SPP*1, STAB1*, and *TYMP* expression); thus, it seems to largely represent proliferating DCs^35–38^ (Figs. 1g, 2a and 2b). We next examined the dynamic change of myeloid cells during GCPM. C1-DC and C2-DC proportions decreased in the G3 Group compared to the G1-G2 Groups (C1-DC, G3 vs G1: *p* = 0.0315, G3 vs G2: *p* = 3.3e-3; C2-DC, G3 vs G1: *p* = 0.0602, G3 vs G2: *p* = 0.0141), whereas C3-DC proportions (G3 vs G1: *p* = 0.3040, G3 vs G2: *p* = 0.5258) did not obviously change (Fig. 1h), indicating that the proportion of cDC2 cells that are proliferating is not related to GC stage. The proportion of monocyte-like DCs in the cDC2 population was significantly higher in the G3 Group than in the G1-G2 Group (G3 vs G1: *p* = 0.0038, G3 vs G2: *p* = 0.0021, Supplementary Fig. 2c). We performed FACS on ascites and peritoneal lavage fluids, and the results validated the existence of both cDC2 and monocyte-like DCs in the peritoneal cavity (Supplementary Fig. 2d). FACS also showed that the proportion of cDC2 cells decreased in ascites (*p* = 0.0355) and the proportion of monocyte-like DCs in cDC2 was higher in ascites than in peritoneal lavage fluids (*p* = 0.0195) (Supplementary Fig. 2e), consistent with our scRNA-seq data. Interestingly, monocyte-like DCs sorted by FACS from ascites showed significant upregulation of transcripts encoding cytokines that can recruit monocytes, including *CCL2, CCL3, CCL4* and *IL1B* (*CCL2*: *p* = 0.0022; *CCL3*: *p* = 0.0205; *CCL4*: *p* = 0.0487; IL1B: *p* = 0.0466) (Supplementary Fig. 2f).

Function scores were applied to explore functional changes in each DC type during GCPM. C1-DC and C4-DC clusters exhibited the highest antigen-presenting capacity among DCs, with no obvious antigen-presenting capacity change in G3 Group, whereas the C2-DC and C3-DC clusters (monocyte-like DCs) exhibited obviously reduced antigen-presenting function score and functional molecule expression in the G3 Group compared to the other stages (Fig. 2a, b and Source Data). The C2-DC and C3-DC cluster showed an increased pro-angiogenic function score and functional molecule expression in the G3 Group during GCPM (Fig. 2a, b). We next explored the dynamic developmental of DCs using Monocle trajectory analysis (Methods). Cell lineage trajectory of DCs showed that the C1-DC and C4-DC clusters were at the origin of the pseudotime trajectory, with the C2-DC cluster at the middle and the C3-DC cluster, characterized by a monocyte-like phenotype, lower antigen-presenting capacity, and higher pro-angiogenic capacity, at the end of the pseudotime trajectory (Fig. 2c). The tumor ecosystem can affect metabolic activity and lead to immune cell dysfunction through metabolic reprogramming, ultimately driving an immunosuppressive, pro-angiogenic, and pro-tumoral phenotype^39–41^. Therefore, we examined metabolic reprogramming in DCs, observing that glycolysis and fatty acid metabolism gradually increased along the differentiation trajectory toward the monocyte-like phenotype, whereas oxidative phosphorylation increased in early and intermediate stages then slightly decreased upon late differentiation (Fig. 2d). We constructed a heatmap to explore dynamic expression changes of genes associated with cellular transitions. C3-DC upregulated mTORC1 activation and E2F signaling pathway-related genes, accompanied by enhanced glycolysis, fatty acid metabolism, oxidative phosphorylation, and cholesterol metabolism (Fig. 2e, f). Immune checkpoint signaling can promote tumor escape from immunosurveillance and regulate cellular metabolism^41,42^. Several immune checkpoint genes (*HAVCR2*, *CD47, LGALS9*, and *CD276*) were gradually upregulated along the DC differentiation trajectory, especially at the late stage, revealing that C3-DC might suppress tumor immunity (Fig. 2e, f). We performed survival analysis of The Cancer Genome Atlas Stomach Adenocarcinoma Cohort (TCGA STAD) using the GEPIA2 tool (http://gepia2.cancer-pku.cn/#index)^43^, and confirmed that the C3-DC cluster gene signature significantly associated with worse prognosis (*p* = 0.0066), making it a potentially useful indicator of adverse clinical outcomes in GCPM (Supplementary Fig. 2g).

We identified three macrophage clusters in the peritoneal ecosystem (Fig. 1f,g). We observed co-existence of M1 and M2 functional phenotypes, indicating their complex function, consistent with previous studies^21,44,45^ (Supplementary Fig. 2h). The C2-Macro cluster preferentially expressed myeloid-derived suppressor cell (MDSC) signature genes including *FCN1 and S100A8*. The C3-Macro cluster preferentially expressed *APOE*, and *TREM2*, resembling an immunosuppressive tumor-associated macrophage phenotype (TAM-like macrophages)^23,44,46^ (Supplementary Fig. 2i,j). We used the TCGA STAD cohort to evaluate the association between the C3-Macro gene signature and prognosis, finding that patients with high C3-Macro gene signature tended to have poor prognosis although there was no statistical significance (median survival time: 24 months vs 60 months; *p* = 0.098, Supplementary Fig. 2k).

### T cell inhibitory states are differentially remodeled in GCPM progression

**Fig. 3:**
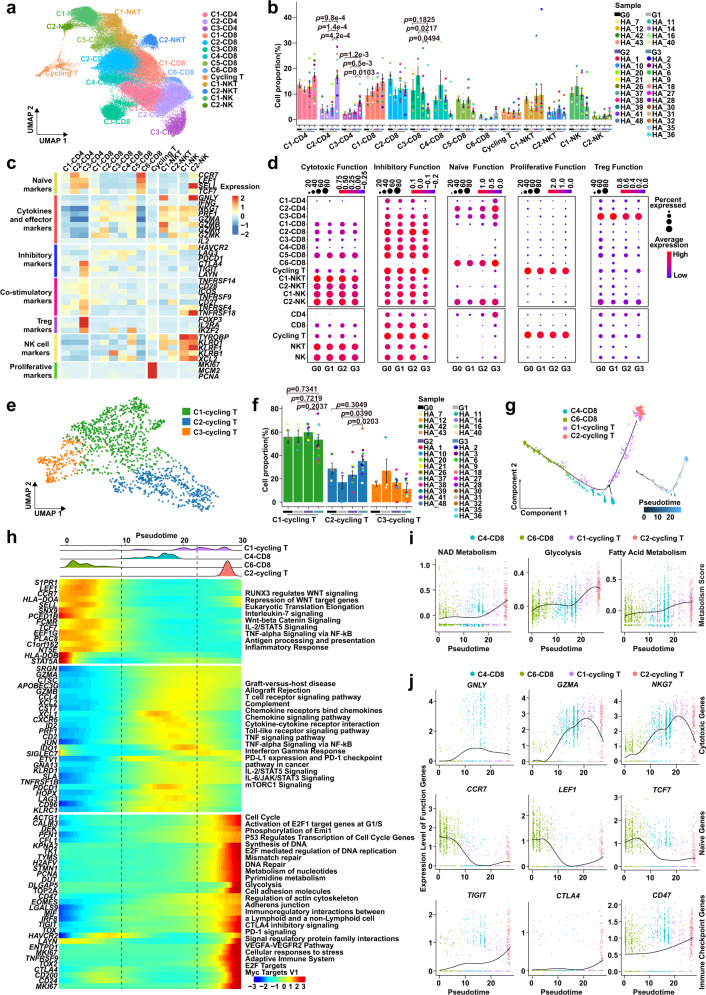
T cell inhibitory states are differentially remodeled in GCPM progression. **a** Uniform Manifold Approximation and Projection (UMAP) plot representing clusters of T/NK cells, colored by cluster. **b** The proportion of T/NK cells clusters in different groups from G0 (*n* = 4 samples), G1 (*n* = 4 samples), G2 (*n* = 10 samples) and G3 (*n* = 12 samples) Groups. Data are presented as mean values ± SEM (error bars); *p*-values are calculated by two-sided unpaired Student’s *t*-test. **c** Heatmap showing expression levels of selected genes of naïve, cytokines and effectors, inhibitory, co-stimulatory, Treg, NK cells, and proliferative markers in each T/NK cell cluster. **d** Dotplot showing the cytotoxic, inhibitory, naïve, proliferative, and Treg function scores of T/NK cells clusters in G0-G3 Groups. Dot size represents percent of expressing cells in each cluster and color represents z-score of normalized mean expression level of selected gene signatures. **e** Uniform Manifold Approximation and Projection (UMAP) plot of three clusters of cycling T cells, colored by cell cluster. **f** Histogram plot indicating the cell proportions of cycling T clusters in G0 (*n* = 4 samples), G1 (*n* = 4 samples), G2 (*n* = 10 samples), and G3 (*n* = 12 samples) Groups. Data are presented as mean values ± SEM (error bars); the *p*-values are calculated by two-sided unpaired Student’s *t*-test. **g** Developmental trajectory of cycling T cells inferred by Monocle 2 analysis, color-coded by cluster (left) and pseudotime (right). Each dot represents a single cell. **h** The distribution of cycling T cells is shown along pseudotime (upper panel), color-coded by T cell clusters. Heatmap showing dynamic expression changes of selected genes and related pathways along pseudotime (lower panel). **i** Curve plots showing dynamic changes of metabolism scores in cycling T cells along pseudotime. Point colors correspond to cell type colors in **g**. **j** Curve plots showing expression changes of function genes related to cytotoxic, naïve, and immune checkpoint genes in cycling T cells along pseudotime. Point colors correspond to cell type colors in **g**. Source data are provided as a Source Data file.

NK cells and especially T cells are key cytotoxic immune cells involved in tumorigenesis and cancer metastasis^47,48^. We conducted unsupervised clustering of all T cells and NK cells, identifying 14 clusters: three CD4^+^ T cell clusters, six CD8^+^ T cell clusters, one proliferative T cell cluster, two NKT cell clusters, and two NK cell clusters (Fig. 3a, b and Supplementary Fig. 3a). We identified the C1-CD4 cluster as effector memory CD4 T cells (*IL7R*-positive and *CCR7*-negative) and the C2-CD4 cluster as naïve CD4 T cells (*CCR7, SELL*, and *LEF1* positive). The C3-CD4 cluster, identified as regulatory CD4 T cells, highly expressed *FOXP3, IL2RA*, and *IKZF2*; co-stimulatory markers *CD28, ICOS*, and *TNFRSF9;* and inhibitory checkpoint genes *CTLA4* and *TIGIT* (Fig. 3c and Supplementary Fig. 3b-d). For CD8 T cells, the C1-CD8 cluster contained effector memory CD8 T cells (*GZMK* and *DUSP2* positive); the C2-CD8 and C5-CD8 clusters were identified as terminally differentiated effector memory/effector CD8 T cells (*GZMA, GZMH, GZMK, CCL4* and *CCL5* positive); the C3-CD8 cluster was identified as mucosal-associated invariant T cells (*SLC4A10* positive); the C4-CD8 cluster co-expressed tissue-resident gene markers (*ITGA1, CD69, CXCR6*, and *CAPG*) and was identified as tissue-resident memory T cells; and the cycling T cluster showed high-proliferative genes (*MKI67, MCM2*, *PCNA*, and *STMN1*) and tissue-resident marker genes (*ITGAE, CD69, CXCR6*, and *CAPG*) (Fig. 3c and Supplementary Fig. 3d, e).

The proportion of C2-CD4 naïve and C3-CD4 Treg clusters increased dramatically and significantly in the G3 Group (C2-CD4, G3 vs G1: *p* = 1.4e-4, G3 vs G2: *p* = 4.2e-4; C3-CD4, G3 vs G1: *p* = 6.5e-3, G3 vs G2: *p* = 0.0103, Fig. 3b). Several well recognized inhibitory immune checkpoint genes such as *HAVCR2, LAG3, PDCD1* and *BTLA* had no expression in most CD4 T cell clusters, whereas *CTLA4* and *TIGIT* had moderate expression in C3-CD4 Treg cells (Fig. 3c and Supplementary Fig. 3c, f). The C3-CD8 mucosal-associated invariant T cells are prevalent in several peripheral tissues and blood and are reported to kill tumor cells^49,50^. The C3-CD8 mucosal-associated invariant T cells cluster proportion decreased in the G3 Group compared to the G1 (*p* = 0.0217) and G2 Groups (*p* = 0.0494), and the cytotoxic function category was inhibited in early-stage disease (G1-G3 Group), demonstrating a rapid response to change in the peritoneal ecosystem^51,52^ (Fig. 3b-d).

We observed a proliferative cycling T cluster characterized by high *MKI67, MCM2* and *PCNA* expression in the peritoneal ecosystem (Fig. 3a, c and Supplementary Fig. 3d, e). These cells exhibited low expression of cytotoxic (*GNLY, IFNG*, and *PRF1*) and naïve (*CCR7* and *SELL*) genes and high expression of inhibitory markers (Fig. 3d and Supplementary Fig. 3e). The cytotoxic function score and corresponding marker genes *GZMA* and *GZMH* were reduced in the G3 Group compared to the G0-G2 Groups (Fig. 3d and Supplementary Fig. 3g). Therefore, the proliferative cycling T cluster may exhibit a dysfunctional status and resemble a naïve phenotype, consistent with previous reports^21,53^. Gene Set Enrichment Analyses (GSEA) indicated the cycling T cluster was enriched in genes involved in cell cycle, glycolysis, and DNA replication, likely preparing the materials and energy required for proliferation, and negatively enriched in cytotoxicity genes (Supplementary Fig. 3h).

We investigated the composition of the proliferative cycling T cell cluster and found that it comprised of heterogeneous cell clusters containing two CD8 cell clusters (C1-cycling T and C2-cycling T) and one NKT cell cluster (C3-cycling T) (Fig. 3e and Supplementary Fig. 3i). The C2-cycling T cell cluster proportion was increased in the G3 Group compared to the G1 (*p* = 0.0390) and G2 Groups (*p* = 0.0203) (Fig. 3f). Using Monocle trajectory analysis to explore the developmental trajectory of peritoneal cycling T cells, we found that the developmental trajectory started with the C6-CD8 naïve cluster, passed through the C4-CD8 and C1-cycling T cell clusters, then culminated in the C2-cycling T cell cluster (Fig. 3g,h). Metabolic transitions are essential for CD8 T cell survival, proliferation, differentiation, and activation in anti-tumor immunity^41,54^. We observed that glycolysis, fatty acid metabolism, and NAD metabolism were obviously up-regulated alongside developmental trajectory, consistent with previous reports^39,41,55^ (Fig. 3g,i). Proliferative C2-cycling T cells highly expressed proliferative genes (*MKI67, STMN1*, and *PCNA*) and gained partial naïve function (*CCR7, LEF1*, *TCF7* and *CCR7*), leading to reduced cytotoxic function (*GNLY, GZMA*, and *NKG7*) and increased inhibitory function (*TIGIT, CD47*, and *CTLA4*) (Fig. 3h,j). Therefore, the C2-cycling T cluster may represent an exhausted and dysfunctional stage, consistent with recent single-cell studies^44,53,56^.

### Therapy-induced evolution of monocyte-like DC and cycling T cell immune phenotype

**Fig. 4:**
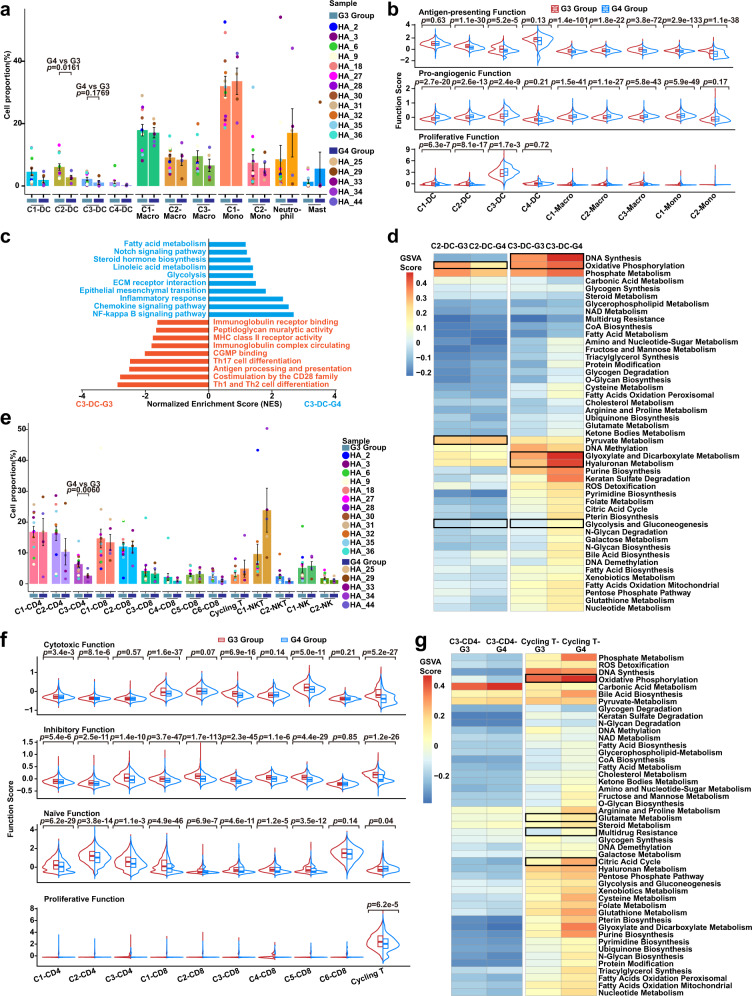
Therapy-induced evolution of monocyte-like DC and cycling T cell immune phenotype. **a** Histogram plot showing cell proportions of myeloid cell clusters in the G3 (*n* = 12 samples) and G4 Groups (*n* = 5 samples) colored by cell type. DC, dendritic cell; Macro, macrophage; Mono, monocyte. Point colors correspond to samples. Data are presented as mean values ± SEM (error bars); *p*-values are calculated by two-sided unpaired Student’s *t*-test. **b** Split violin plots showing the antigen-presenting, pro-angiogenic, and proliferative function scores of myeloid cells in the G3 (*n* = 12 samples, red) and G4 Groups (*n* = 5 samples, blue) groups. Box represents median ± interquartile range; *p-*values are calculated by two-sided unpaired Wilcoxon test. **c** Gene Set Enrichment Analyses (GSEA) analysis showing distinct enrichment pathways of C3-DC in the G3 (red) and G4 Groups (blue). Bar chart showing normalized enrichment scores (NES) of specific pathways. **d** Heatmap plot representing the activity of metabolism pathways of C2/C3-DCs in the G3 and G4 Groups. Color indicates the activity score of each metabolism pathway calculated by Gene Set Variation Analysis (GSVA) analysis. **e** Histogram plot representing the cell proportion of T/NK clusters in the G3 (*n* = 12 samples) and G4 Groups (*n* = 5 samples), colored by cell type. Point colors correspond to samples. Data are presented as mean values ± SEM (error bars); *p-*values are calculated by two-sided unpaired Student’s *t*-test. **f** Split violin plots showing the cytotoxic, inhibitory, naïve, and proliferative function scores of T cells in the G3 (*n* = 12 samples, red) and G4 Groups (*n* = 5 samples, blue). Box represents median ± interquartile range; *p*-values are calculated by two-sided unpaired Wilcoxon test. **g** Similar to **d**, the metabolism pathway activity score of C3-CD4 and cycling T cells in the G3 and G4 Groups shown in a heatmap plot. Pathway activity scores were calculated by Gene Set Variation Analysis (GSVA) analysis. Source data are provided as a Source Data file.

Cells of the tumor ecosystem can exhibit therapy-induced evolution of biological heterogeneity involving cell state transitions and cellular plasticity^23,57,58^. We observed that the C2-DC cluster decreased (*p* = 0.0161) following therapy (G4 Group; Fig. 4a). We next explored functional changes during therapy-induced evolution. Therapy caused C3-DC cells (shown earlier to be a late-stage population in GC) to gain proliferative and pro-angiogenic capacity, and their antigen-presenting capacity significantly decreased (Fig. 4b). Analysis with the “SCENIC” R package suggested that the C3-DC cluster had distinctive transcriptional factor (TF) activity between the G3 and G4 Groups, characterized by *PPARG*, *MLX* and *CEBPA* activation (Supplementary Fig. 4a). By comparing differentially expressed genes (DEGs) of the C3-DC cluster between the G3 and G4 Groups, we confirmed that the C3-DC cluster in the G4 Group was characterized by high expression of genes involved in immunosuppression (*APOE* and *GPNMB*) and pro-angiogenesis (SPP*1*) (Supplementary Fig. 4b). Furthermore, C3-DC cells significantly upregulated inflammatory factors *CCL2, CCL3* and *CCL4* in the G4 Group, which may recruit inflammatory monocytes, monocyte-like DCs, neutrophils and TAM-like macrophages into the peritoneum through their corresponding receptors *CCR1, CCR2*, and *CCR5*, leading to a pro-angiogenic and immunosuppressive ecosystem^59–61^ (Supplementary Fig. 4b,c). GSEA analysis found that C3-DC cells in the G4 Group highly expressed genes involved in inflammatory response, NF-kappa B signaling, chemokine signaling, epithelial-mesenchymal transition, and Notch signaling (Fig. 4c). Conversely, C3-DC cells in the G3 Group highly expressed genes involved in antigen processing and presentation, MHC-II receptor activity, and T helper cell differentiation (Fig. 4c).

Cell state and function transitions are often accompanied by heterogenic metabolic patterns^62–64^. We used Gene Set Variation Analysis (GSVA) to assess metabolic activity in monocyte-like DC clusters between the G3 and G4 Groups. The C3-DC cluster upregulated oxidative phosphorylation and glycolysis after therapy, accompanied by hyaluronan metabolism and purine metabolism in the G4 Group (Fig. 4d). In contrast, the C2-DC cluster exhibited decreased mitochondrial function, characterized by downregulated oxidative phosphorylation and fatty acid mitochondria oxidation and increased glycolysis and pyruvate metabolism (Fig. 4d). Systemic therapy significantly decreased the proportion of C3-CD4 Treg cells (*p* = 0.0060) that had increased during GCPM with a reduced inhibitory and naïve function, suggesting an improving immunosuppressive status (Fig. 4e, f). The cycling T cell cluster exhibited distinct TF activity in the G3 and G4 Groups, characterized by *PPARG, ZBTB14, MLX, BATF*, and *CEBPA* activation (Supplementary Fig. 4d). The cycling T cell cluster in the G4 Group had impaired adaptive immune function compared to the G3 Group, including a low expression of cytotoxic genes (*NKG7, GZMA, GZMH*, and *PRF1*) and MHC molecules (*HLA-DRA, HLA-DPA1*, and *HLA-C*) (Supplementary Fig. 4e). Notably, cycling T cells acquired higher plasticity (*ALDH1A1, SOX9*, and *SOX4*) and lower proliferation (*STMN1*) in the G4 Group (Supplementary Fig. 4e). Furthermore, function score analysis indicated that cycling T cells exhibited a reduced cytotoxic/proliferative function and an increased naïve function under anti-tumor therapy (G4 Group), suggesting that cycling T cell function may be silenced in response to the stress of therapy and that this transition may be a self-protection mechanism against antitumor drugs (Fig. 4f). The cycling T cell cluster also expressed genes associated with drug metabolism (*MGST1, GSTP1, NME1* and *NME2*)^65–67^ (Supplementary Fig. 4e). Metabolism analysis indicated that cycling T cells undergo obvious metabolic reprogramming during therapy, with upregulated oxidative phosphorylation, citric acid cycle, and glutathione metabolism possibly associating with drug metabolism (Fig. 4g).

### Immune checkpoint evolution of monocyte-like DCs and cycling T cells

**Fig. 5:**
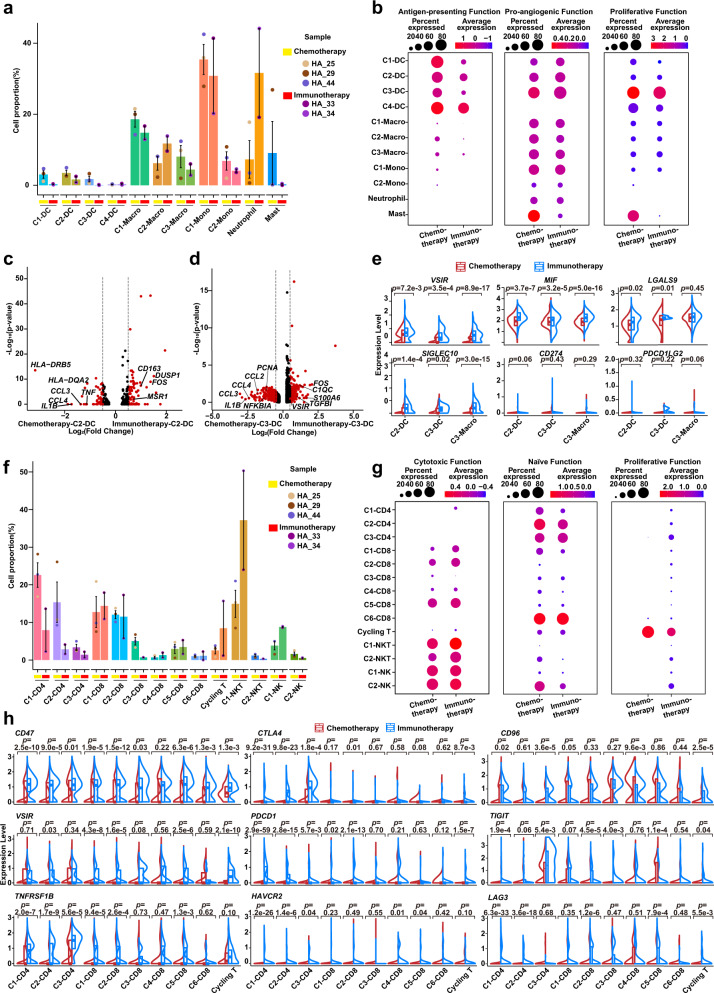
Immune checkpoint evolution of monocyte-like DC and cycling T cells. **a** Cell proportions of myeloid cell clusters after Chemotherapy (*n* = 3 samples) and Immunotherapy (*n* = 2 samples) shown in histogram plot. DC, dendritic cell; Macro, macrophage; Mono, monocyte. Histogram colors correspond to cell clusters; point colors correspond to samples. Data are presented as mean values ± SEM (error bars). **b** Dotplot showing the antigen-presenting, pro-angiogenic, and proliferative function scores of myeloid cell clusters in Chemotherapy (*n* = 3 samples) and Immunotherapy (*n* = 2 samples) Groups. Dot size represents percent of expressing cells in each cluster, and color represents mean expression level of selected gene signatures. **c**, **d** Volcano plots showing differentially expressed genes (DEGs) of C2-DC (**c**) and C3-DC (**d**) between Chemotherapy (three patients) and Immunotherapy (two patients) Groups. **e** Split violin plots showing expression levels of immune checkpoints of C2/C3-DC and C3-Macro in Chemotherapy (*n* = 3 samples, red) and Immunotherapy (*n* = 2 samples, blue) Groups. The comparisons and statistical analyses are conducted between cell clusters (three cell clusters: C2-DC, C3-DC, and C3-Macro) of Chemotherapy and Immunotherapy groups, and the total cells number of all clusters are >3. Box represents median ± interquartile range; *p*-values are calculated by two-sided unpaired Wilcoxon test. **f** The same histogram plot as in **a** for T/NK clusters in Chemotherapy (*n* = 3 samples) and Immunotherapy (*n* = 2 samples) Groups. Histogram colors correspond to cell clusters; point colors correspond to samples. Data are presented as mean values ± SEM (error bars). **g** Dotplot showing the cytotoxic, naïve, and proliferative function scores of T/NK cell types in Chemotherapy (three patients) and Immunotherapy (two patients) Groups. Dot size represents percent of expressing cells in each cluster, and color represents z-score of normalized mean expression level of selected gene signatures. **h** The same split violin plots as in (**e**) for T/NK cell types in Chemotherapy (*n* = 3 samples, red) and Immunotherapy (*n* = 2 samples, blue) Groups. The comparisons and statistical analyses are conducted between T cell clusters (10 cell clusters: C1-3 CD4, C1-6 CD8, and Cycling T) of Chemotherapy and Immunotherapy groups, and the total cells number of all clusters are >3. Box represents median ± interquartile range; *p*-values are calculated by two-sided unpaired Wilcoxon test. Source data are provided as a Source Data file.

Cancer immunotherapy can remodel the phenotype of immune cells infiltrating into solid tumors^68^, but its impact in the peritoneal ecosystem remains unclear. Therefore, we explored immune checkpoint evolution in immune cells by comparing ascites samples from immunotherapy (two patients) and chemotherapy (three patients) (Supplementary Table 1). We found inhibited overall antigen-presenting capacity for all myeloid cells but largely comparable pro-angiogenic capacity (Supplementary Fig. 5a). Specifically, C2-DC and C3-DC proportions decreased in immunotherapy compared to chemotherapy (Fig. 5a). In cell function analysis, the C2-DC cluster downregulated antigen-presenting capacity without significant changes to pro-angiogenic capacity, whereas the C3-DC cluster downregulated both antigen-presenting and pro-angiogenic capacities in immunotherapy (Fig. 5b). By comparing C2-DC DEGs between the immunotherapy and chemotherapy groups, we found C2-DC cells in the immunotherapy group had lower-expressed genes associated with MHC molecules (*HLA-DRB5, HLA-DQA2*) (Fig. 5c). Notably, C2-DC cells following immunotherapy were negatively enriched for pro-inflammatory phenotype (*CCL4, IL1B, CCL3* and *TNF*), with high expression of anti-inflammatory associated genes (*CD163* and *MSR1*) (Fig. 5c). Similarly, C3-DC and C3-Macro clusters exhibited an anti-inflammatory phenotype under immunotherapy, negatively enriching for the NF-kappa B pathway and pro-inflammatory phenotype, suggesting a widespread shift to an anti-inflammatory phenotype for monocyte-like DCs and TAM-like macrophages with lower *CCL2, CCL3, CCL4, IL1B, TNF*, and *NFKBIA* (Fig. 5d and Supplementary Fig. 5b,c). To better understand the role of these monocyte-like DCs in immunotherapy, we examined immune checkpoint expression patterns. *CD274* (PD-L1) and *PDCD1LG2* (PD-L2), the two ligands for the PD-1-related immune checkpoint, were rarely expressed in monocyte-like DCs and TAM-like macrophages. Other immune checkpoint genes such as *VSIR, MIF, LGALS9*, and *SIGLEC10*, which can induce T/NK cell immune checkpoints and subsequently inhibit their cytotoxicity^69–72^, were expressed in C2-DC and C3-DC clusters and increased in immunotherapy compared with chemotherapy (Fig. 5e).

We next explored immune phenotype changes in peritoneal-infiltrating T cells during immunotherapy. C2-CD4 naïve and C3-CD4 Treg cluster proportions decreased in immunotherapy compared to chemotherapy (Fig. 5f), indicating that immunotherapy may reduce immunosuppressive T cell clusters. In addition, immunotherapy caused increased expression of co-stimulatory marker genes *CD27, CD69*, and *TNFRSF14* (Supplementary Fig. 5d), consistent with a previous report^73^. Cell function analysis indicated that the percent of CD8 T cells with high cytotoxic function slightly increased, although the overall cytotoxic function score of CD8 T cells in immunotherapy was comparable to the chemotherapy group, and the naïve function score of CD4 T cells in the immunotherapy group decreased compared to the chemotherapy group (Supplementary Fig. 5e). Further function score analysis showed that immunotherapy did not obviously enhance T cell cytotoxicity, but could increase the percent of CD8 T cells with high cytotoxic function (Fig. 5g). However, immunotherapy significantly induced upregulation of several immune checkpoints. The C3-CD4 Treg cluster in the immunotherapy group preferentially expressed immune checkpoint genes *CTLA4, TIGIT, CD47, CD96*, and *TNFRSF1B* after immunotherapy, and *VSIR, CD47, CD96*, and *TNFRSF1B* were upregulated in the cycling T cluster after immunotherapy (Fig. 5h).

### High-plasticity GC evolves to high-proliferative GC through a conserved cellular program

**Fig. 6:**
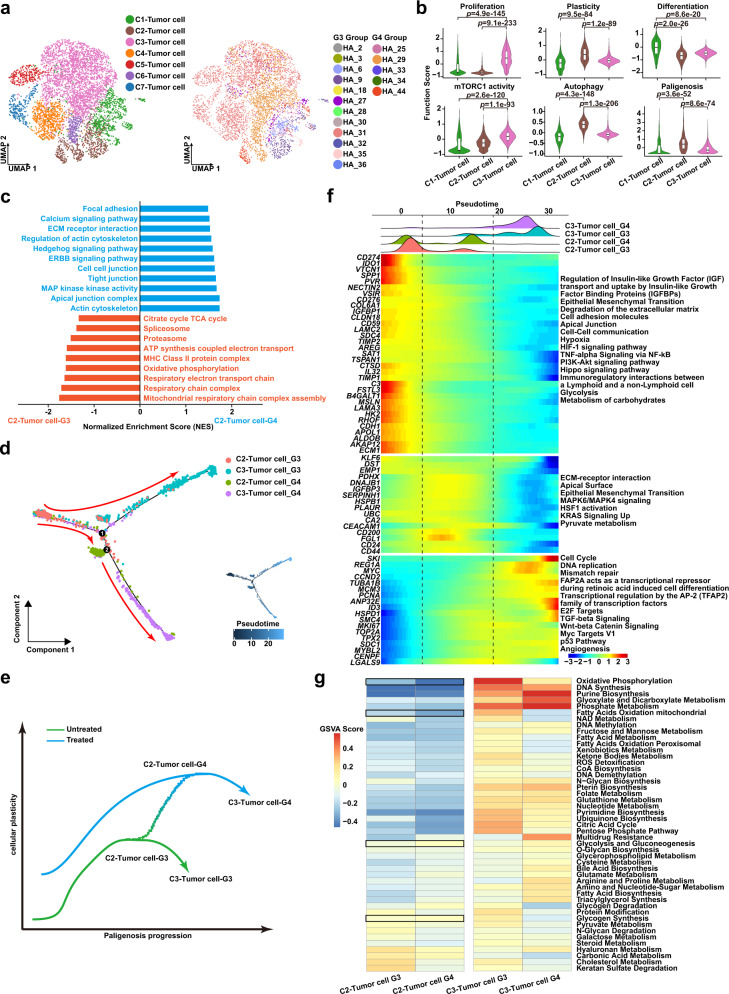
High-plasticity GC evolves to high-proliferative GC through a conserved cellular program. **a** Uniform Manifold Approximation and Projection (UMAP) visualization of tumor cells by cell types (Left, colors correspond to cell clusters) and samples (Right, colors correspond to samples). **b** Violin plot showing function scores of proliferation, plasticity, differentiation, mTORC1, autophagy, and paligenosis in tumor cells (C1-Tumor cell: *n* = 713 cells; C2-Tumor cell: *n* = 549 cells; C3-Tumor cell: *n* = 2545 cells). Box represents median ± interquartile range; whiskers represent 1.5x interquartile range; *p-*values are calculated by two-sided unpaired Wilcoxon test. **c** Gene Set Enrichment Analyses (GSEA) analysis showing distinct enrichment pathways of C2-Tumor cells in the G3 (red) and G4 Groups (blue). Bar chart showing the normalized enrichment score (NES) of specific pathways in specific tumor cells. **d** The trajectory plot of C2/C3-tumor cells in the G3 and G4 Groups (left), and the transition trajectories along pseudotime (right) in a two-dimensional state-space inferred by Monocle 2 analysis. **e** Two-dimensional schematic diagram showing cellular plasticity changes in C2/C3-tumor cells in the G3 and G4 Groups along paligenosis progression. **f** The 3-phase distribution of C2/C3-tumor cells along pseudotime color-coded by cell cluster (upper panel). Heatmap showing dynamic expression changes of genes and related pathways of C2/C3-tumor cells in the G3 and G4 Groups along pseudotime (lower panel). **g** Heatmap plot indicating the activity of metabolism pathways in C2/C3-tumor cells between the G3 and G4 Groups. Color represents the activity score of metabolism pathways calculated by Gene Set Variation Analysis (GSVA) analysis. Source data are provided as a Source Data file.

All epithelial cells were divided into 7 clusters. We observed that C1-3 tumor clusters were shared by almost all GC cases, while C4-7 tumor clusters were present only in a single case (Fig. 6a). We analyzed tumor characteristics such as proliferation, differentiation, and plasticity in clusters C1-3 and found that the C1-tumor cluster was highly differentiated, the C2-tumor cluster was highly plastic, and the C3-tumor cluster was highly proliferative (Fig. 6b). Recently a conserved program for cellular plasticity in epithelial cells was defined, termed paligenosis. Paligenosis occurs via 3 stages: Stage1, massive activation of autophagy and lysosomal activity as mTORC1 is extinguished; Stage2, re-expression of progenitor or embryonic markers without mTORC1 expression; Stage3, induction of high mTORC1 with cell cycle entry^19,20,74–77^, consistent with a previous study^78^. Previously, paligenosis has been shown as the process differentiated cells use to return to the progenitor state in precancerous states like gastric metaplasia. We reasoned that GC, which largely arises from metaplastic lesions, might maintain plasticity in response to therapy by continuing to use paligenosis. The C2-tumor cluster had high autophagy (*ATG5, MAP1LC3B*), high plasticity (*SOX4, CD164*) and low mTORC1 activity (*RPS6, MLST8*) (Supplementary Fig. 6a), characteristics of early paligenosis (stages 1-2). The early paligenosis markers *DDIT4* and *ATF3* were also upregulated (Supplementary Fig. 6a). The C3-tumor cluster had high mTORC1 activity (*MLST8, RPS6*) and high proliferation (*MKI67, MCM2*), characteristics of late paligenosis when cells re-enter the cell cycle (stage 3)^74,76,77^ (Fig. 6b and Supplementary Fig. 6a). We applied pseudotime trajectory analysis to the C1-3 tumor clusters and found a continuous developmental trajectory between the C2-tumor and C3-tumor clusters, while the C1-tumor had strong heterogeneity and hence seemed to follow a separate developmental mechanism, as they were evenly distributed along the trajectory (Supplementary Fig. 6b). C1-tumor cells may be akin to differentiated cells in a tissue prior to paligenosis, as C1-tumor cells have increased “Differentiation” and decreased “Proliferation” and “Plasticity” phenotypes.

We next analyzed therapy-induced evolution of GC cells during GCPM. In normal tissue, paligenosis of differentiated cell is induced by injury or inflammation that causes mature, mitotically quiescent cells to reprogram and reenter the cell cycle^75,79^. Thus, if paligenosis was the mechanism tumor cells were using to increase proliferation, we would expect therapy to induce reprogramming of tumor cell clusters. All clusters changed significantly following therapy, especially plasticity and paligenosis characteristics of the C2-tumor cluster (Supplementary Fig. 6c, d). GSEA analysis confirmed that C2-tumor cluster plasticity was further enhanced after therapy (G4 Group) as characterized by Hedgehog, ERBB pathway activation, and cell polarization remodeling (Fig. 6c). In further trajectory analysis, we discovered that part of the C2-tumor cluster in the G3 Group develops into the C3-tumor cluster in the G3 Group, with the other part developing into the highly-plastic C2-tumor cluster in the G4 Group. At the terminal end of the cell trajectory, C2-tumor cells from the G4 Group lose some plasticity and gain proliferative ability and develop into the C3-tumor cluster from the G4 Group. The whole development trajectory is a continuous evolution of cell plasticity leading to proliferation (Fig. 6d–f). We analyzed expression changes of genes associated with cell transitions. In the middle of the trajectory where the C2-tumor-G4-Group cluster is enriched, cells were characterized by epithelial-mesenchymal transition, a program often seen in cells gaining plasticity and therapy resistance^80–83^ (Fig. 6f). At the end of cell trajectory, where the C3-tumor-G4-Group cluster is enriched, cells were characterized by cell cycle entry and Wnt pathway activation, demonstrating that C3-tumor cells in the G4 Group maintain considerable pluripotency while gaining proliferative ability (Fig. 6f). Both C2-tumor and C3-tumor clusters in the G4 Group showed metabolic reprograming and increased multi-drug resistance. C2-tumor cells in the G4 Group were further characterized by decreased mitochondrial function (oxidative phosphorylation and fatty acid oxidation) and increased glycolysis and glycogen metabolism (Fig. 6g).

### Autophagy inhibition blocks paligenosis and induces apoptosis in GC PDOs

**Fig. 7:**
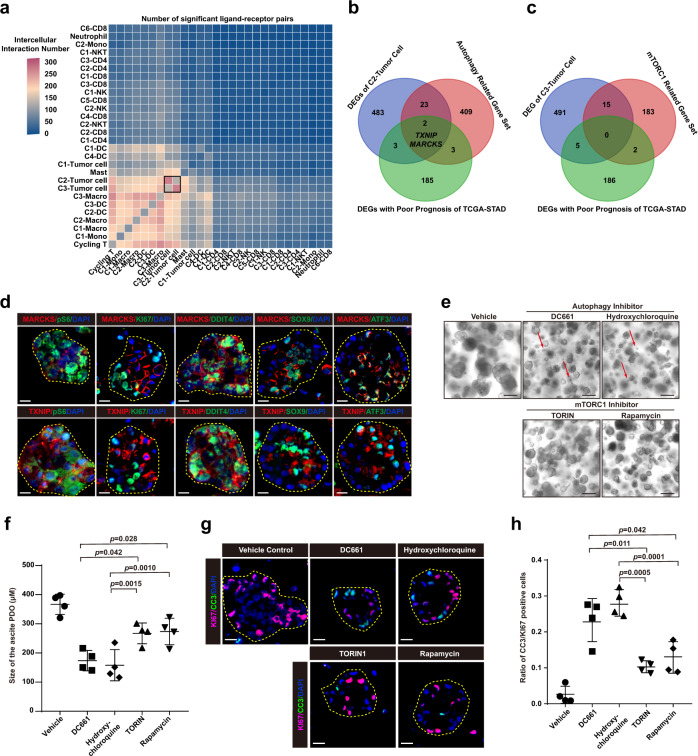
Autophagy inhibition blocks paligenosis and induces apoptosis in GC PDOs. **a** Heatmap showing the intercellular interaction by CellPhoneDB analysis. Color represents the number of significant ligand-receptor pairs among different cell subtypes. DC, dendritic cell; Macro, macrophage; Mono, monocyte. **b**, **c** Venn diagram showing the number of overlapping signature genes between differentially expressed genes (DEGs) of C2-tumor cell, poor prognostic DEGs of The Cancer Genome Atlas Stomach Adenocarcinoma (TCGA-STAD) database, and autophagy-related gene sets (**b**) and mTORC1-related gene sets (**c**). **d** Immunofluorescence staining for MARCKS (red, upper panel) and TXNIP (red, lower panel), early paligenosis markers DDIT4 (green) and ATF3 (green), late paligenosis markers KI67 (green) and pS6 (green), progenitor-related marker SOX9 (green), and nuclei marker DAPI (blue) in 15th generation patients-derived organoids from ascites. Scale bar, 100 μm. The experiment was repeated with 4 independent experiments, with similar results. **e** Fifteenth generation organoids generated as clones from single cells dissociated from 14th generation organoids after addition of inhibitors or vehicle when single cell suspensions were replated. Red arrows indicate application of autophagy and mTORC1 inhibitors promoting organoids death. Scale bar, 400 μm. The experiment was repeated with four independent experiments, with similar results. **f** Quantification of the size of 15th generation organoids as in (**e**) after 7-day treatment with autophagy or mTORC1 inhibitors (*n* = 4 independent experiments). Each datapoint represents the mean of mean values of organoids in all wells. Every well included the means of 25+ counts. Data are presented as mean values ± SEM (error bars); *p*-values are calculated by one-way ANOVA with Tukey post hoc test. **g** Immunofluorescence staining for apoptosis marker CC3 (green), proliferation marker KI67 (pink), and nuclei marker DAPI (blue) in 15th generation organoids as in (**e**) after 7-days treatment with autophagy or mTORC1 inhibitors. Scale bar, 100 μm. **h** The ratio of CC3/KI67 positive cells as in (**g**) (*n* = 4 independent experiments). Data are presented as mean values ± SEM (error bars); *p*-values are calculated by one-way ANOVA with Tukey post hoc test.

To further clarify cellular regulation during GCPM, we analyzed cell communication among all cell clusters in the peritoneal ecosystem. We found complex communication among GC and immune cells. Surprisingly, the strongest cell communication was between C2 and C3 tumor cells (Fig. 7a and Supplementary Fig. 7a, b). To decode the molecular network regulating C2 and C3 tumor cells, we looked for intersections between DEGs of C2 tumor cells, autophagy-related genes, and genes related to GC patient prognosis in the TCGA STAD database. Only two transcripts, *MARCKS* (Myristoylated Alanine Rich Protein Kinase C Substrate) and *TXNIP* (Thioredoxin Interacting Protein) were present in all three gene sets in the C2 tumor cell cluster (Fig. 7b and Supplementary Fig. 7c, d). No intersecting genes were discovered among C3 tumor cell DEGs, a published set of mTORC1-related genes, and genes related with GC patient prognosis in TCGA (Fig. 7c). PDOs comprise effective tools for genetic evolution studies, biomarker identification, and drug screening for cancer patients^84–86^. We cultured PDOs from ascites samples from four advanced GC patients with GCPM (Supplementary Table 3). As shown in Supplementary Fig. 7e, GC PDOs maintained the growth patterns and differentiation of the primary GC. MARCKS and TXNIP protein expression did not correlate with expression of late (Stage 3) paligenosis markers pS6 and KI67^19,77^; however, they were co-expressed with early paligenosis markers DDIT4 and ATF3 as well as SOX9 (which is induced by Stage 2), indicating that MARCKS and TXNIP are enriched in early paligenosis^74,76^ (Fig. 7d). We treated ascites PDOs with autophagy or mTORC1 inhibitors, as autophagy induction and mTORC1 activation are hallmarks of early and late paligenosis, respectively, and both are required for cells undergoing paligenosis to eventually re-enter the cell cycle^19,77^. Both autophagy and mTORC1 inhibitors significantly decreased the growth of ascites PDOs as measured by organoid size (Fig. 7e, f). The autophagy inhibitors DC661 and hydroxychloroquine were more effective at inhibiting the growth of ascites PDOs compared with mTORC1 inhibitors TORIN1 and rapamycin (Fig. 7e, f). We discovered that only DC661 or hydroxychloroquine induced organoid death, indicating that autophagy and mTORC1 inhibitors hinder PDOs growth via different mechanisms (red arrow in Fig. 7e). This result was consistent with our previous studies that autophagy blockage causes cell death in paligenosis stages 1-2, whereas mTORC1 blockage simply inhibits cell cycle entry in paligenosis stage 3^19,77^. To further analyze the features of organoid death upon treatment with autophagy and mTORC1 inhibitors, we stained PDOs treated with autophagy or mTORC1 inhibitors for CC3 (cleaved caspase3) and KI67. DC661 and hydroxychloroquine significantly induced the apoptosis of ascites PDOs, increasing the ratio of CC3/KI67 positive cells compared with untreated control and mTORC1 inhibitors treated PDOs, confirming that autophagy inhibition caused failed paligenosis and subsequently induce apoptosis (Fig. 7g,h).

To examine the influence of autophagy and mTORC1 inhibitors on peritoneal metastasis in vivo, three groups of mice were all intraperitoneally injected with PDOs and then treated with PBS, hydroxychloroquine, or rapamycin. Positron Emission Tomography (PET) displayed that both hydroxychloroquine and rapamycin clearly inhibited peritoneal metastasis, and hydroxychloroquine was more effective (Supplementary Fig. 7f,g), consistent with the PDO results in vitro.

## Discussion

GCPM has poor prognosis and high mortality with poor therapeutic outcomes^2–4^. The study of GPCM is complicated since the complexity of the peritoneal ecosystem and the mechanisms of GCPM remain to be deciphered. Here, we used single-cell RNA sequencing to comprehensively characterize the peritoneal ecosystem. Our analysis reveals distinct and dynamic changes within the ascites ecosystem during GCPM and illustrates the evolution of peritoneal cells induced by chemotherapy and immunotherapy at single-cell resolution.

Antigen presentation is the main function of DCs^87–89^, and we found that DC clusters exhibited slightly increased antigen-presenting capacity in the G1 Group compared to the G0 Group and then decreased antigen-presenting capacity in the G2 Group compared to the G1 Group, consistent with their higher cell proportions among the G0-G2 Groups, suggesting that early-stage disease may have an effect on DC proportion and function. This supports the current viewpoint that primary tumors can modify distant ecosystems to form pre-metastatic niches before tumor metastasis^90,91^. The DC cluster variations across different disease stages, particularly in cDC2, indicate the complexity of the DC-mediated tumor immune response caused by GCPM. Monocyte-derived DCs can impede differentiation and IFN production in CD4 T cells^92^. The proliferative C3-DC cluster expressed lower MHC-I and MHC-II levels, indicative of a monocyte-like phenotype with reduced antigen-presenting capacity and increased pro-angiogenic functions and cell proportion during GCPM. Developmental trajectory analysis confirmed that the C3-DC cluster featured immunosuppressive, pro-angiogenic and proliferative capacity. Altogether, based on their dynamic changes in cell proportion and function, monocyte-like DCs contribute to a unique peritoneal immune ecosystem, aiding in formation of an immunosuppressive and pro-angiogenic ecosystem favorable for GCPM.

T cells represent key cytotoxic immune cells during tumorigenesis and cancer metastasis^48^. Both C2-CD4 naïve and C3-CD4 Treg clusters showed increased cell proportion and naïve function score during GCPD, whereas the C1-CD4 T cell cluster was more prone to a naïve phenotype in GCPM than in non-GCPM without obvious changes in cell proportion. Notably, expression of genes involved in naïve function such as *SELL* and *CCR7* increased along with disease state, but genes involved in inhibitory checkpoints (i.e., *CTLA4* and *TIGIT*) were not obviously affected. Therefore, these CD4 clusters either increased their naïve function or their immunosuppressive cell proportion, implying that both non-functional and immunosuppressive cells become enhanced in the peritoneal ecosystem during GCPM. Moreover, CD8 cluster cytotoxic function was attenuated in peritoneal tumor immunity in GCPM. Notably, the C2-CD8 cluster was characterized by low expression of cytokines/chemokines (*CCL3*, *CCL4L2, XCL1*, and *XCL2*), which might hinder infiltration of effector CD8 T/NK cells and DCs into the peritoneum for their anti-tumor functions^93–95^. Thus, T cell immune function already begins to deplete prior to GCPM, with significant depletion in GCPM and an obviously increased naïve/immunosuppressive phenotype, eventually promoting GCPM by inhibiting peritoneal immunity.

Paligenosis is a conserved cellular program for cells to undergo plastic changes respond to damage and maintain homeostasis^19^. It has previously been characterized in multiple normal tissues and cells. For successful paligenosis, cells require stress response proteins such as DDIT4, ATF3 and IFRD1, whose actions modulate critical cell energy and survival networks centered on mTORC1 and P53^77^. During gastric tumorigenesis, there is evidence that pre-cancerous metaplasia is a normal, wound-healing, regenerative response that is proceeded by paligenosis. However, when paligenosis is abnormal, paligenotic cells with DNA damage are not eliminated or stalled until DNA repair is complete. Such cells can then accumulate DNA damage and undergo malignant transformation, ultimately leading to diffuse GC initiation^75,76^. Once GC is established via aberrant paligenosis, GC cells may maintain this abnormal paligenosis propensity, which they induce in response to stress of various kinds: either endogenous due to hypoxia/starvation from tumor overgrowth of blood supply or exogenous due to chemotherapy or immunotherapy. We presume that GC cells can use a modified paligenosis program (and its associated metabolic/mTORC1 modulating processes) to evade immune cells and therapy and promoting invasion and metastasis as cancer progresses.

In the current study, we noticed GC cells shifted from a high-plasticity state (early paligenosis with autophagy) to a high-proliferative state (late paligenosis with strong mTORC1 activation) in untreated GCPM patient ascites, with a cell trajectory consistent with the paligenosis program. Previous studies reported that mTORC1 controls the adaptive transition of quiescent cells in G0 to re-enter the cell cycle in response to injury-induced signals^78^. Accordingly, we observed that the highly plastic GC cells in the G3 Group evolved to gain even higher plasticity upon systemic therapy in the G4 Group. The simultaneous appearance of these different high-plasticity clusters is consistent with the highly heterogenous nature of GC^17,96,97^. Therapy-resistance is usually achieved by acquiring high-plasticity states through increased expression of stemness-related genes^98,99^. Within the plasticity response, such stemness-related genes (like *SOX9*) might be induced via paligenosis, the first stage of which is a large upregulation of autophagy and lysosomes.

Tumors are composed of heterogeneous cell populations that exhibit varying degrees of functional and genetic heterogeneity^100,101^. Cancer stem cells (CSCs) were proposed four decades ago, and are defined as rare cell populations with unlimited self-renewal potential, driving tumorigenesis, clonogenicity, metastasis and drug resistance, and maintaining tumor microenvironment^101–103^. Interestingly, in this study, we found two different populations in the peritoneal cavity microenvironment of GCPM (C2-Tumor cell and C3-Tumor cell clusters) that could functionally serve as CSCs. One cluster (C2 Tumor cells) expressed stem-related genes (*SOX4* and *CD164*); the other (C3-Tumor cells) expressed proliferative genes (*MKI67* and *MCM2*). Cell trajectory analysis showed that the two clusters could transition from C2-Tumor cell to C3-Tumor cell clusters, undergoing inactivation of stem-related signal pathways and activation of proliferative signal pathways. Thus, CSC potential may be maintained by plasticity via a paligenosis-like program. Importantly, paligenosis is a stepwise, specific program with multiple cellular pathways involved. Considering tumor plasticity from a paligenosis perspective thus may open many avenues to design antitumor therapies and/or augment existing therapy. Promisingly, we found that targeting paligenosis using autophagy and mTORC1 inhibitors reduced proliferation of PDOs from ascites by inducing apoptosis.

Tumor infiltrating immune cells feature immunosuppressive and pro-tumorigenic functions and play distinct and complex roles in tumor ecosystems^104,105^. Tumor cells with pluripotent and plastic characteristics can self-renew to propagate tumorigenesis and metastasis^106–108^. Immune cells support tumor growth and metastasis and also remodel the tumor ecosystem to maintain a suitable niche^109^. For example, TAM-like macrophages have been reported to promote cancer stem cell-like characteristics via cytokine pathways^110,111^. Our cell–cell interaction analysis highlighted intercellular interactions between monocyte-like DCs and cycling T clusters and between C2 tumor cells and C3 tumor cells in the peritoneal ecosystem. Therefore, we speculate that proliferative monocyte-like DCs and cycling T cells maintain tumor cell plasticity in the peritoneal cavity via ligand-receptor communication pathways. In our study, SPP*1* and *HGF* were indicated to mediate crosstalk between proliferative C3-DC and tumor cells to maintain stem cell ability in tumor cells via SPP1-CD44 and HGF-CD44 ligand-receptors^112–114^. Proliferative cycling T cells may also play critical roles in maintaining tumor cell plasticity in GCPM via SPP1-CD44^112^. Thus, to effectively prevent and treat GCPM, immunotherapy strategies must consider the unique immunological characteristics and stemness of peritoneal infiltrating tumor cells and their intercellular interactions with immune cells.

Studying the ecosystems of ascites caused by other ascites-producing conditions will be helpful to provide a crucial direction for the further study of ascites of gastric cancer. A published study indicates that the myeloid cells and lymphocytes in hepatocellular-carcinoma-related ascites have distinct origins and are predominantly linked to primary tumor and peripheral blood origins, respectively^44^. Ascites caused by high-grade serous ovarian cancer (HGSOC) is known to be associated with drug resistance and a poor prognosis^115^, and the ascites ecosystem harbors several distinct cell clusters including immune cells, epithelial cells and cancer-associated fibroblasts^116^. Interestingly, the molecular subtypes of HGSOC mainly reflect the tumor ecosystem composition (the abundance of immune infiltrates and fibroblasts) rather than distinct subsets of malignant cells, and inflammatory reprogramming of tumor cells depends on an intact ascites microenvironment, reflecting an endogenous property of the tumor cells^116^. Since the immune characteristics of peripheral blood are quite different from those of tumors, and sampling ascites via paracentesis is far easier and safer than tissue biopsy, these results provide a direction for the detection of tumor status by sampling ascites instead of peripheral blood or biopsy tissue. Further study should explore the specific ecosystem characteristics of ascites of gastric cancer by comparing the ascites caused by other different ascites-producing conditions.

In conclusion, our study describes different cell developmental lineage trajectories in the peritoneal ecosystem and provides evidence to better understand the mechanism of GCPM, aiding exploration of effective target strategies for GCPM therapy.

## Methods

### Patient samples and ethics statement

Seventeen samples of malignant ascites fluid were collected from patients pathologically diagnosed with GC and 18 peritoneal lavage fluid samples were collected from GC patients without GCPM or from benign hysteromyoma patients from four medical centers: the First Hospital of China Medical University, the Sheng Jing Hospital of China Medical University, the Liaoning Cancer Hospital & Institute, and the People’s Hospital of Liaoning Province. Early gastric cancer is limited to the mucosa or submucosa (pT1), with a very low risk of distant metastasis and is amenable to endoscopic resection, while advanced gastric cancer refers to tumors that invade the muscularis propria and beyond (pT2 or higher) recommended radical gastrectomy with lymphadenectomy^117–122^. Of these collected samples, four control peritoneal lavage fluid samples were taken from four benign hysteromyoma patients (negative controls, G0 Group); peritoneal lavage fluid from four early GC patients (G1 Group); peritoneal lavage fluid from 10 advanced GC patients (G2 Group); ascites from 12 untreated GC patients with diagnosed GCPM (G3 Group) and ascites from five GC patients with diagnosed GCPM following systemic therapy (G4 Group). Of the G4 group, three and two patients were treated with chemotherapy and anti-PD-1/PD-L1immunotherapy, respectively. Some ascites samples were obtained under laparoscopic exploration due to GCPM. Detailed clinical, pathological, and therapeutic history information are summarized in Supplementary Table 1. This study was performed following the ethical guidelines of the Declaration of Helsinki and was approved by the Research Ethics Committee of China Medical University and these hospitals (Shenyang, China). Informed consent was obtained from all enrolled patients before collection of samples and clinical information.

### Sample collection and single-cell suspension processing

Ascites fluids and peritoneal lavage fluids were immediately transported to our laboratory on ice following drainage. Liquid samples were aliquoted into 50-ml centrifuged tubes through 70-um cell strainers (Cat# 352350, BD FALCON) and centrifuged at 300 g for 5 min at 4 °C. After supernatant removal, the remaining cell pellet was resuspended in red blood cell lysis buffer (Cat# R1010, Solarbio) and incubated on ice for 5 min to remove red blood cell contamination. Phosphate-buffered saline (PBS; Cat# SH30256.01, HyClone) was used to quench the red blood cell lysis buffer and then the cell suspension was centrifuged at 300 g for 5 min at 4 °C to pellet the cells. The above process of red blood cell lysis was repeated until no red blood cells were visible. The resulting cell pellet was washed twice with PBS then resuspended in sorting buffer (PBS containing 2% fetal bovine serum [FBS; Cat# DT-100-S, DearyTech]). Cell concentration and viability were analyzed using Countstar Automated Cell Counter (Model# Rigel S2, ALIT Life Sciences) with acridine orange/propidium iodide (AO/PI) double staining assay (Cat# CS2-0106-5ML, Nexcelom Bioscience). Single-cell suspensions with over 85% viability were used for scRNA-seq assay. Generally, the entire process from sample collection to single-cell suspension loaded on the 10x Chromium Controller microfluidic device (10x Genomics) was completed within 2-4 h.

### Single-cell RNA library preparation and sequencing

Nanoliter-scale Gel Beads-in-emulsion (GEM) generation & barcoding, reverse transcription, complementary DNA (cDNA) amplification, and 3' gene expression dual index library construction steps were conducted according to the manufacturer’s instructions of the 10x Genomics Chromium single cell 3' platform (10x Genomics). Briefly, single-cell GEM generation was performed on a 10x Chromium Controller (Mode l# GCG-SR-1, 10x Genomics) using Chromium Next GEM Single Cell 3ʹ Reagent Kit v3.1 (Cat# PN-1000268, 10x Genomics) and Chromium Next GEM Chip G Single Cell Kit (Cat# PN-1000120, 10x Genomics). Concentrations of single cell suspensions were adjusted to 800-1200 cells/μL (measured by CountStar), and then about 10000 cells per sample were loaded on each channel of the 10x Genomics Chromium system (GEM generation & barcoding) per sample. The cells were partitioned into GEM to achieve single cell resolution. Captured single cells were lysed and the released RNAs were barcoded through reverse transcription in individual GEMs. Reverse transcription of GEMs was performed using a Thermal Cycler (Model# 9902, Applied Biosystems) at 53 °C for 45 min, 85 °C for 5 min, then held at 4 °C. cDNA was PCR amplified to generate sufficient mass for library construction and cDNA quality was assessed using an Agilent 4200 TapeStation system (Agilent Technologies). Finally, barcoded cDNA libraries were sequenced on an Illumina Novaseq6000 sequencer using a pair-end 150 bp (PE150) reading strategy (CapitalBio Technology, China). To reduce batch effects among samples, all cDNA libraries were constructed using the same reagent kit and protocol.

### Ascites PDOs culture and treatment

PDOs were derived from ascites drainage samples from four patients with GCPM. Ascites drainage was filtered with a 70-um cell strainer then centrifuged at 300 g for 5 min. Pellets were resuspended into single-cell suspensions with PBS and incubated with red blood cell lysis buffer for 5 min on ice, then centrifuged again at 300 g for 5 min. Finally, the cells were embedded in Matrigel, seeded onto pre-warmed 24-well culture plates, and cultured in Advanced DMEM/F12 medium (GIBICO) with 50% L-WRN conditioned medium supplemented with 10 mM HEPES (GIBICO), 10 mM Nicotinamide (Sigma), 1X N2 (GIBICO), 1X B27 (GIBICO), 1X Glutamax (GIBICO), 1.25 mM N-Acetylcysteine (Sigma-Aldrich), 50 ng/mL EGF (Peprotech), 200 ng/mL FGF10 (Peprotech), 10 nM gastrin (R&D Systems), and 1X Primocin (Invivogen). 10 uM ROCK inhibitor (Y-27632, R&D Systems) was provided for the first generation of PDOs to prevent anoikis. PDOs were overlaid with culture medium and incubated at 37 °C in humidified air containing 5% CO_2_. Culture medium was changed every 3 days. For passaging, PDOs were collected by mechanically dissociating the Matrigel, centrifuged at 150 g for 5 min, incubated with pre-warmed TrypLE express for 7 min, then pipetted about 100 times. All PDOs were trypsinized to single cells under a light microscope then transferred into new matrigel with culture medium and plated in pre-warmed 24 well-plates, incubated at 37 °C in humidified air containing 5% CO_2_. PDOs were passaged every 7 days until at least the 15th generation. PDOs were treated with culture media and inhibitors including Hydroxychloroquine Sulfate (1uM), DC661 (4uM), TORIN1 (2uM), or Rapamycin (400 nM) for 7 days before analysis. Culture medium and drugs were changed every 3 days. To embed PDOs, culture media was removed, PDOs were washed in DPBS on ice for 15 mins, then PDOs were fixed in 10% formalin on ice for 2 h. Finally, PDOs were moved to 75% ethanol at 4 °C overnight, mounted in 3% agar, and embedded in paraffin.

### Patient-derived organoid xenograft (PDOX) animal experiments

NOD/SCID mice were purchased from Beijing Vital River Laboratory Animal Technology (Beijing, China). Ascites organoids were digested into single cells using TrypLE. An amount of 1 × 10^6^ cells in 0.2 mL Advanced DMEM/F12 medium with 2% FBS were intraperitoneal injected to the 6-week-old female NOD/SCID mice. Two weeks after the cells were injected, mice were treated with rapamycin and hydroxychloroquine every 3 days for 3 weeks. For rapamycin group, mice were treated with 3 μg/g rapamycin (LC Laboratories) in 0.25% Tween-20, 0.25% polyethylene glycol in PBS every 3 days. For hydroxychloroquine sulfate group, mice were treated with 30 μg/g hydroxychloroquine sulfate (Selleck) in PBS every 3 days. After 5 weeks of cell injection, tumor-bearing mice were injected intravenously with 18-fluorodeoxyglucose (18F-FDG) (8.32 ± 0.92MBq) via the tail-vein. For best contrast, PET imaging performed after 30 min metabolism. Commercial software MadicPet was used to reconstruct PET images. The maximum standardized uptake value (SUV_max_) was measured for semi-quantitative analysis. After the 18F-FDG PET scan, mice were sacrificed and peritoneal tumors were isolated and embedded in paraffin. All animal experiments were performed in accordance with the National Institutes of Health Guide for Care and Use of Laboratory Animals, the ARRIVE guidelines, and the institutional ethical guidelines (China Medical University Animal Studies Committees). Mice were sacrificed by euthanasia when the tumor size reached the limitation (maximal tumor diameter ≥ 10 mm).

### Immunofluorescence staining

Organoid microtome sections underwent a standard deparaffinization protocol with xylene and rehydration then were antigen retrieved in sodium citrate buffer (2.94 g sodium citrate, 500 uL Tween 20, pH 6.0) using a pressure cooker. Sections were blocked in 1%BSA, 0.3% Triton X-100 in PBS for 60 min. Primary antibodies were applied overnight at 4 °C, see Supplementary Table 4. For immunofluorescence staining, organoid slides were washed in PBS and incubated with Alexa-fluor (Invitrogen) secondary antibodies at room temperature for 60 min, then 4',6-Diamidino-2-phenylindole (DAPI) was used to detect nuclei. Slides were washed in PBS and mounted using ProLong Gold antifade mountant with DAPI (Molecular Probes) and stored at 4 °C. All immunofluorescence images of organoids were captured with citation3 (BioTek instruments, Inc.). All experiments were performed at least three times by the same scientific researcher. Detailed information of antibody application and dilution is shown in Supplementary Table 4.

### Enzyme-linked immunosorbent assay (ELISA)

The concentrations of cytokines of ascites and peritoneal lavage fluids were measured by ELISA (R&D Systems®). Briefly, samples were centrifuged at 300 g for 5 min at 4 °C to remove the cell pellet. The detailed experiment processes were performed according to the manufacturer’s instructions of the ELISA kits. Finally, the concentrations of cytokines were measured via microplate reader (Infinite M200 Pro, TECAN) capable of measuring absorbance at 450 nm, with the correction wavelength set at 540 nm. ELISA data were collected using Tecan i-control Software (version 1.11, TECAN).

### Flow cytometry (fluorescence-activated cell sorting, FACS) of monocyte-like dendritic cells

Single-cell suspension was obtained after filtration and centrifugation. The cells for FACS were stained with Fluorophore-conjugated antibodies containing CD45-eFluor (Cat# 69-0459-42, eBioscience™), CD3-FITC (Cat# 11-0037-42, eBioscience™), CD56-FITC (Cat# 11-0566-42, eBioscience™), CD19-FITC (Cat# 11-0199-42, eBioscience™), CD1c-APC (Cat# 331524, BioLegend), CD163-PE (Cat# 12-1639-42, eBioscience™), CD14-BV421(Cat# 563743, DB Biosciences) on ice for 30 min. After washing twice with FACS buffer, the cells were stained using 7-AAD Viability Staining Solution (Cat# 00-6993-50, eBioscience™) on ice 5 min. FACSAriaIII (BD Biosciences) was used for FACS. FACS data were collected using BD FACS Diva Software (version 8.0.2, BD), and data were analyzed with FlowJo software. Detailed information of antibody application and dilution is shown in Supplementary Table 4.

### Single-cell RNA-seq data processing

Raw scRNA-seq data were processed using Cell Ranger Software (version 4.0.0, 10x Genomics) obtained from the 10x Genomics official website (https://support.10xgenomics.com/single-cell-gene-expression/software/overview/welcome) for demultiplexing, barcode processing, read alignment to GRCh38 human reference genome, single-cell 3' gene counting, and generation of feature (gene)-barcode expression matrix. The preliminary filtered data generated by Cell Ranger were used for downstream analysis. Quality of cells was assessed based total UMI counts per cell, total detected genes per cell, and proportion of mitochondrial genes per cell. Low-quality cells were filtered following these criteria: (1) cells with <200 genes; (2) cells with <800 UMI counts or ranked in the top 1% of UMI counts; (3) cells with >20% mitochondrial gene count. Genes detected in less than three cells were also excluded from downstream analyses. Subsequently, the “DoubletFinder” R package was used to predict and remove potential doublets^123^. Library size normalization was performed to obtain a normalized barcode-count matrix using the “NormalizeData” function in “Seurat” R package based on the gene counts of each cell^124^. Briefly, size factors of each cell were computed by dividing the total gene UMI counts in that cell by a scale factor of 10000 (Size Factor = total gene UMI counts/10000), and then normalized gene UMI counts (GUC_normalized_) for each cell were computed by dividing the gene UMI counts by the size factor for that cell (GUC_normalized_ = Gene UMI Counts/Size Factor), which was a TPM-like values. Finally, normalized gene expression values were quantified as log_2_(GUC_normalized_ + 1) for subsequent downstream analyses.

### Multiple scRNA-seq dataset integration and batch correction

This study included different samples of ascites fluid and peritoneal lavage fluid from benign hysteromyoma patients and different stages of GC to explore the landscape and dynamic change of the peritoneal ecosystem during GCPM. Samples were from different donors (G0-G4 Groups), and the whole experiment of single-cell sequencing was performed several times due to sample collection. Thus, batch effects were an important factor that could not be ignored. To integrate multiple scRNA-seq datasets into a shared space from different groups for unsupervised clustering and reduce batch effects, the harmony integration algorithm was used on the PCA space, which has been reported to reduce experimental and technical batch effects while preserving biological variation and the continuous state of developmental cells rather than erroneously clustering cells into discrete groups^125^. The Harmony algorithm has been widely used to integrate multiple single-cell RNA-seq datasets^44,126–128^.

### Dimensionality reduction, unsupervised cell clustering, and visualization

The Seurat v3 R package was used for data scaling, highly variable gene selection, dimensionality reduction, unsupervised clustering, and visualization by using the normalized filtered feature-barcode expression matrix^124^. In brief, the matrix was scaled using the “ScaleData” function in the Seurat R package, and highly variable genes, which could preserve major biology variation, were identified for subsequent principal component analysis (PCA) using the “FindVariableGenes” and “RunPCA” functions in “Seurat” R package. An appropriate number of principal components were used based on the PCA results, and specific resolution parameters were selected for further unsupervised graph-based clustering to determine an optimal number of cell clusters using the “FindNeighbors” and “FindClusters” functions. For visualization of graph-based cell clustering, Uniform Manifold Approximation and Projection (UMAP) was applied with the “RunUMAP” function.

### Differential expressed gene analysis, cell annotation, and cell re-clustering

To identify cluster-specific markers genes, differential expressed gene (DEG) analysis between the corresponding cluster compared with all other clusters was performed using the Seurat “FindMarkers” function with default parameters of the Wilcoxon rank-sum test. Significant DEGs were defined as |log_2_(Fold Change)| > 0.50 and False Discovery Rate (FDR) < 0.01. Cell clusters were annotated following the following steps: (1) Cell type was automatically annotated using the “singleR” R package (https://github.com/dviraran/SingleR)^129^, with unbiased cell type recognition by leveraging reference transcriptomic datasets of pure cell types to infer the cell of origin for each single cell independently; (2) We summarized sets of well-known marker genes for each cell types; (3) Cell annotation by the “singleR” function was manually verified or corrected based on expression of known marker genes and based on the top 50 most upregulated genes in each cluster; (4) Cells simultaneously expressing two or more sets of marker genes of cell types were labeled as doublets or multiplets and excluded from the downstream analysis.

In the first round of cell clustering and cell annotation, major cell types including T cells, NK cells, macrophages/monocytes, dendritic cells, neutrophils, mast cells, tumor cells, B cells, plasma cells, fibroblasts, and mesothelial cells were identified. To further analyze major cell types, re-integration, re-clustering, and re-annotation were conducted on myeloid cells (macrophages/monocytes, dendritic cells, neutrophils, and mast cells), T cells/NK cells, and tumor cells.

In addition, to measure correlations among different cells, cluster marker genes were first chosen based on DEGs then the Pearson correlation coefficient was computed between the average expression profiles of cluster marker genes in cells. This was presented by hierarchical clustering heatmap. Correlations between certain cell types in different groups were compared to explore potential function changes along disease progression.

### Single-cell CNV analysis

For cell clusters labeled as epithelial cells, inferCNV (https://github.com/broadinstitute/infercnv) was applied to compute somatic large-scale chromosomal copy number variations (CNV), such as gains or deletions of entire chromosomes or large segments of chromosomes, in each single cell to identify malignant epithelial cells^130,131^. InferCNV sorted all analyzed genes by their genomic locations and applied a moving average of 101 genes within each chromosome to estimate initial chromosomal CNVs (CNV_initial_) in each cell and at each analyzed gene using the following CNV equation:

${{CNV}}_{k}\left({{gene}}_{i}\right)={\sum }_{j=i-50}^{i+50}{E}_{k}({{gene}}_{j})/101$, where CNV_k_ (gene_i_) is the CNV value of gene i in cell k, gene_*j*_ is the gene j in cell k, and E_k_(gene_j_) is the normalized expression of gene j in cell k.

To verify the reliability of the CNV results, 1000 T cells and 1000 B cells were added to the epithelial cells as “Observations” cell inputs (as internal negative validation). In addition, another 1000 T cells and 1000 B cells were used as a set of reference “normal” cells. The inferred CNV values of “Observations” cells was initially the CNV value of “Observations” cells subtracted from the initial CNV value of “normal”cells in the corresponding gene area (inferred CNV = CNV_observations_initial_ - CNV_normal _initial_). The inferCNV used a Hidden Markov Model (HMM) Model (i6 HMM model) to predict CNV level and implemented a Bayesian Network Latent Mixture Model to identify the posterior probabilities of alteration status in each cell and whole CNv region to correct the results. The i6 HMM model was a six-state CNV score model to predict the following CNV levels: 0: complete loss; 0.5: loss of one copy; 1: neutral; 1.5: addition of one copy; 2.0: addition of two copies; 3.0: >2 copies. A heatmap was generated according to CNV score per cell. T cells and B cells were clustered together and labeled as “non-tumor cells”, whereas all epithelial cells were clustered together separately from T and B cells and labeled as “tumor cells”. The inferCNV results showed that all epithelial cells in G3 and G4 Group were malignant tumor cells.

### Trajectory inference analysis

The Monocle2 algorithm was applied to infer potential cell lineage trajectories between diverse cell phenotypes (https://github.com/cole-trapnell-lab/monocle-release; http://cole-trapnell-lab.github.io/monocle-release/)^132,133^. Cell lineage trajectories of CD8 + T and dendritic cell were inferred and characterized at the single-cell level. A UMI counts matrix was used as the input. The “newCellDataSet” function was used to create a CellDataSet object with parameter “expressionFamily=negbinomial.size()” following the Monocle2 tutorial. Based on significant DEGs, dimensionality reduction was performed using the DDRTree algorithm. The cell lineage trajectory based on cell cluster and pseudotime was then inferred with the default parameters of Monocle2 after dimensionality reduction and cell ordering, then visualized with the “plot_cell_trajectory” function. Following cell trajectory, DEGs along pseudotime (named “pseudotime-dependent genes”) were found using the “differentialGeneTest” function. Then “plot_genes_in_pseudotime” and “plot_pseudotime_heatmap” functions were used to visualize dynamic changes of pseudotime-dependent gene expression along pseudotime, and significant pseudotime-dependent genes were also visualized with a heatmap. Similarly, the BEAM test of Monocle 2 was applied to identify branch-dependent gene expression dynamically expressed along each branch, and significant branch-dependent genes were visualized with a heatmap.

### Definition of gene signatures and cell function scores

To define cell function scores, top upregulated genes between cell clusters based on DEG analysis and published well-known functional genes were used. Nine gene signatures were identified, including cytotoxic, inhibitory, naïve, proliferative, and Treg function gene signatures for T/NK cells, antigen-presenting, pro-angiogenic, phagocytotic, pro-inflammatory, anti-inflammatory, and proliferative function gene signatures for myeloid cells, M1 and M2 gene signatures for macrophage cells, and autophagy, plasticity, paligenosis, mTORC1, proliferation, and differentiation function gene signatures for tumor cells. Detailed gene signatures are listed in Supplementary Data 1. Defining the average expression level of gene signatures as cell function and cell status has been widely used in single-cell analysis^21,134,135^, thus we defined the average expression of these gene signatures after z-score transformation as cell function and cell state for these cell types, and the original expression of each gene was measured by log_2_(GUC_normalized_+1). Comparison of cell function scores between different groups were tested by two-sided unpaired Wilcoxon test.

### Metabolism pathway activity analysis

To estimate metabolism pathway activities for specified cell types, metabolism pathway gene signatures were obtained from a curated database and PathCards (https://pathcards.genecards.org/)^136,137^. Metabolism pathways with <3 genes were excluded to make the analysis robust. Finally, the “GSVA” R package was used to perform Gene Set Variation Analysis (GSVA) to estimate metabolism pathway activities (http://www.bioconductor.org/packages/release/bioc/html/GSVA.html)^138^.

### Pathway enrichment analysis

To investigate differences in biological states and pathways between different cell types, Gene Set Enrichment Analysis (GSEA), a computational method that determines whether an a priori defined set of genes shows statistically significant, concordant differences between two biological states, was performed (https://www.gsea-msigdb.org/gsea/index.jsp)^139^. Gene sets for GSEA were obtained from the Molecular Signatures Database (MSigDB) (https://www.gsea-msigdb.org/gsea/msigdb/index.jsp). KEGG, GO, Reactome, and Hallmark pathway enrichment analysis were performed^140–143^.

### Cell–cell interaction analysis

CellPhoneDB was used to explore cell–cell interactions between different cell types^144^. Briefly, for each gene in each cell type, the average expression value of the gene and the percentage of cells expressing the gene were calculated. Potential receptor-ligand interactions between cell types were inferred based on the expression of receptors in one cell type and ligands in the other, and then the cell type labels of all cells were randomly permuted 1000 times to test the statistical significance of the estimated receptor-ligand interaction. The intensity of receptor-ligand interactions was assessed based on expression of the ligand-receptor pairs in two cell types.

### Transcription factor analysis

Transcription factor (TF) activity analysis was performed using the “SCENIC” R package which could infer co-expression regulatory networks between TFs and candidate target genes from scRNA-seq dataset (https://github.com/aertslab/SCENIC)^145^. The input matrix was the normalized feature-barcode expression matrix. The SCENIC workflow was performed as described previously^145^. Briefly, the workflow had three steps: (1) “GENIE3” R package identified potential TF targets based on co-expression; (2) “RcisTarget” R package performed the TF-motif enrichment analysis and identified direct targets; and (3) “AUCell” R package scored the activity of the direct targets on single cells.

### PDO size and immunofluorescence staining quantification

PDO size was measured by counting at least 25 randomly sampled whole fields to prevent experimental bias. All treatments were quantified across four samples of organoids and each sample was repeated four times by the same scientific researcher. Quantification of CC3/KI67 cells was done by counting at least 10 randomly sampled whole fields. Statistics for comparing between multiple groups were analyzed using one-way ANOVA with Tukey post hoc test to determine significance. Data were generally expressed as mean ± standard error of mean (SEM). Single datapoints plotted were almost always means of 25+ counts, so means on plots were means of means. *p* < 0.05 was considered statistically significant for interpretation in the text. All PDOs sizes were measured in Image J (v1.52a, NIH), all statistics were performed in GraphPad Prism (v8.0.1, GraphPad Software).

### Statistical analysis

The two-sided unpaired Wilcoxon test, Pearson’s correlation test, log-rank test, and Student’s *t*-test were used in this study. The comparisons and statistical analyses in split violin plots are conducted between cell clusters of different groups, and the total cells number of all clusters are >3. A two-sided *p*-value < 0.05 was considered statistically significant. All statistical analyses were conducted using R version 4.0.5 (R Foundation for Statistical Computing, Vienna, Austria), and Python version 3.9.0 (Python Software Foundation). Detailed descriptions on statistical analysis are described in the results section and Figure legends.

### Reporting summary

Further information on research design is available in the Nature Portfolio Reporting Summary linked to this article.

## Supplementary information

Supplementary Info

Reporting Summary

Description of Additional Supplementary Files

Supplementary Data 1

## Data Availability

The single-cell RNA sequencing data generated in this study have been deposited in the Genome Sequence Archive for Human (GSA-Human) database under accession code HRA002712, under controlled access. Access will be granted for academic use only. Access can be granted by request from the corresponding author (Zhen-Ning Wang, josieon826@sina.cn). Access will be granted within 2 weeks and there is no limitation on duration of access. The processed single-cell RNA sequencing data are available at Source Data file. The detailed data for Figures and Supplementary Figures are summarized in the Source Data file. Source data are provided with this paper.

## Source data

Source Data

## Code availability

This study did not generate any unique code. All software and algorithms used in this study are freely or commercially available and are listed in the Methods section.

## References

[1] Sung H (2021). Global Cancer Statistics 2020: GLOBOCAN estimates of incidence and mortality worldwide for 36 cancers in 185 countries. CA: Cancer J. Clin..

[2] Kus T (2021). Prediction of peritoneal recurrence in patients with gastric cancer: a multicenter study. J. Gastrointest. Cancer.

[3] Nakamura M (2019). Conversion surgery for gastric cancer with peritoneal metastasis based on the diagnosis of second-look staging laparoscopy. J. Gastrointest. Surg..

[4] Mizrak Kaya D (2018). Risk of peritoneal metastases in patients who had negative peritoneal staging and received therapy for localized gastric adenocarcinoma. J. Surg. Oncol..

[5] Takahashi Y (2022). Real-world effectiveness of nivolumab in advanced gastric cancer: the DELIVER trial (JACCRO GC-08). Gastric Cancer.

[6] White MG (2020). Factors associated with resection and survival after laparoscopic HIPEC for peritoneal gastric cancer metastasis. Ann. Surg. Oncol..

[7] Newhook TE (2019). Laparoscopic hyperthermic intraperitoneal chemotherapy is safe for patients with peritoneal metastases from gastric cancer and may lead to gastrectomy. Ann. Surg. Oncol..

[8] Saito T (2015). Preferential HER2 expression in liver metastases and EGFR expression in peritoneal metastases in patients with advanced gastric cancer. Gastric Cancer.

[9] Du S (2021). Anoikis resistant gastric cancer cells promote angiogenesis and peritoneal metastasis through C/EBPbeta-mediated PDGFB autocrine and paracrine signaling. Oncogene.

[10] Ye G (2020). Nuclear MYH9-induced CTNNB1 transcription, targeted by staurosporin, promotes gastric cancer cell anoikis resistance and metastasis. Theranostics.

[11] Natsume M (2020). Omental adipocytes promote peritoneal metastasis of gastric cancer through the CXCL2-VEGFA axis. Br. J. Cancer.

[12] Zhang J (2021). Single-cell transcriptomics provides new insights into the role of fibroblasts during peritoneal fibrosis. Clin. Transl. Med..

[13] Si M (2019). Inhibition of hyperglycolysis in mesothelial cells prevents peritoneal fibrosis. Sci. Transl. Med..

[14] Pan G (2020). Discovering biomarkers in peritoneal metastasis of gastric cancer by metabolomics. OncoTargets Ther..

[15] Ohzawa H (2020). Exosomal microRNA in peritoneal fluid as a biomarker of peritoneal metastases from gastric cancer. Ann. Gastroenterol. Surg..

[16] Hu Y (2019). Malignant ascites-derived exosomes promote peritoneal tumor cell dissemination and reveal a distinct miRNA signature in advanced gastric cancer. Cancer Lett..

[17] Wang R (2021). Single-cell dissection of intratumoral heterogeneity and lineage diversity in metastatic gastric adenocarcinoma. Nat. Med..

[18] Wang R (2020). Multiplex profiling of peritoneal metastases from gastric adenocarcinoma identified novel targets and molecular subtypes that predict treatment response. Gut.

[19] Willet SG (2018). Regenerative proliferation of differentiated cells by mTORC1-dependent paligenosis. EMBO J..

[20] Brown JW, Cho CJ, Mills JC (2022). Paligenosis: cellular remodeling during tissue repair. Annu. Rev. Physiol..

[21] Sun Y (2021). Single-cell landscape of the ecosystem in early-relapse hepatocellular carcinoma. Cell.

[22] Dong R (2020). Single-cell characterization of malignant phenotypes and developmental trajectories of adrenal neuroblastoma. Cancer Cell.

[23] Maynard A (2020). Therapy-induced evolution of human lung cancer revealed by single-Cell RNA sequencing. Cell.

[24] Tirosh I (2016). Dissecting the multicellular ecosystem of metastatic melanoma by single-cell RNA-seq. Science.

[25] Braoudaki M (2022). Chemokines and chemokine receptors in colorectal cancer; multifarious roles and clinical impact. Semin Cancer Biol..

[26] Lopez-Cotarelo P, Gomez-Moreira C, Criado-Garcia O, Sanchez L, Rodriguez-Fernandez JL (2017). Beyond chemoattraction: multifunctionality of chemokine receptors in leukocytes. Trends Immunol..

[27] Griffith JW, Sokol CL, Luster AD (2014). Chemokines and chemokine receptors: positioning cells for host defense and immunity. Annu Rev. Immunol..

[28] Brown CC (2019). Transcriptional basis of mouse and human dendritic cell heterogeneity. Cell.

[29] Dutertre CA (2019). Single-cell analysis of human mononuclear phagocytes reveals subset-defining markers and identifies circulating inflammatory dendritic cells. Immunity.

[30] Mellman I (2013). Dendritic cells: master regulators of the immune response. Cancer Immunol. Res..

[31] Binnewies M (2019). Unleashing type-2 dendritic cells to drive protective antitumor CD4(+) T cell immunity. Cell.

[32] Salmon H (2016). Expansion and activation of CD103(+) dendritic cell progenitors at the tumor site enhances tumor responses to therapeutic PD-L1 and BRAF inhibition. Immunity.

[33] Segura E (2013). Human inflammatory dendritic cells induce Th17 cell differentiation. Immunity.

[34] Haniffa M (2012). Human tissues contain CD141hi cross-presenting dendritic cells with functional homology to mouse CD103+ nonlymphoid dendritic cells. Immunity.

[35] Cheng S (2021). A pan-cancer single-cell transcriptional atlas of tumor infiltrating myeloid cells. Cell.

[36] Zhang L (2020). Single-cell analyses inform mechanisms of myeloid-targeted therapies in colon cancer. Cell.

[37] Casazza A (2013). Impeding macrophage entry into hypoxic tumor areas by Sema3A/Nrp1 signaling blockade inhibits angiogenesis and restores antitumor immunity. Cancer Cell.

[38] Toi M, Atiqur Rahman M, Bando H, Chow LW (2005). Thymidine phosphorylase (platelet-derived endothelial-cell growth factor) in cancer biology and treatment. Lancet Oncol..

[39] Xia L (2021). The cancer metabolic reprogramming and immune response. Mol. cancer.

[40] Kaymak I, Williams KS, Cantor JR, Jones RG (2021). Immunometabolic interplay in the tumor microenvironment. Cancer Cell.

[41] Leone RD, Powell JD (2020). Metabolism of immune cells in cancer. Nat. Rev. Cancer.

[42] Chang CH (2015). Metabolic competition in the tumor microenvironment is a driver of cancer progression. Cell.

[43] Tang Z, Kang B, Li C, Chen T, Zhang Z (2019). GEPIA2: an enhanced web server for large-scale expression profiling and interactive analysis. Nucleic Acids Res..

[44] Zhang Q (2019). Landscape and dynamics of single immune cells in hepatocellular carcinoma. Cell.

[45] Azizi E (2018). Single-cell map of diverse immune phenotypes in the breast tumor microenvironment. Cell.

[46] Obradovic A (2021). Single-cell protein activity analysis identifies recurrence-associated renal tumor macrophages. Cell.

[47] Gonzalez H, Hagerling C, Werb Z (2018). Roles of the immune system in cancer: from tumor initiation to metastatic progression. Genes Dev..

[48] Chen DS, Mellman I (2017). Elements of cancer immunity and the cancer-immune set point. Nature.

[49] Melo AM (2019). Mucosal-associated invariant T cells display diminished effector capacity in oesophageal adenocarcinoma. Front. Immunol..

[50] Toubal A, Nel I, Lotersztajn S, Lehuen A (2019). Mucosal-associated invariant T cells and disease. Nat. Rev. Immunol..

[51] Parrot T (2020). MAIT cell activation and dynamics associated with COVID-19 disease severity. Sci. Immunol..

[52] Shaler CR (2017). MAIT cells launch a rapid, robust and distinct hyperinflammatory response to bacterial superantigens and quickly acquire an anergic phenotype that impedes their cognate antimicrobial function: Defining a novel mechanism of superantigen-induced immunopathology and immunosuppression. PLoS Biol..

[53] Zhang Y (2021). Single-cell analyses reveal key immune cell subsets associated with response to PD-L1 blockade in triple-negative breast cancer. Cancer Cell.

[54] Ghesquiere B, Wong BW, Kuchnio A, Carmeliet P (2014). Metabolism of stromal and immune cells in health and disease. Nature.

[55] Ma EH (2019). Metabolic profiling using stable isotope tracing reveals distinct patterns of glucose utilization by physiologically activated CD8(+) T cells. Immunity.

[56] Li H (2019). Dysfunctional CD8 T cells form a proliferative, dynamically regulated compartment within human melanoma. Cell.

[57] Connor AA, Gallinger S (2021). Pancreatic cancer evolution and heterogeneity: integrating omics and clinical data. Nat. Rev. Cancer.

[58] Ushijima T, Clark SJ, Tan P (2021). Mapping genomic and epigenomic evolution in cancer ecosystems. Science.

[59] Korbecki J (2020). CC chemokines in a tumor: a review of pro-cancer and anti-cancer properties of the ligands of receptors CCR1, CCR2, CCR3, and CCR4. Int. J. Mol. Sci..

[60] Vilgelm AE, Richmond A (2019). Chemokines modulate immune surveillance in tumorigenesis, metastasis, and response to immunotherapy. Front. Immunol..

[61] Li X (2017). Targeting of tumour-infiltrating macrophages via CCL2/CCR2 signalling as a therapeutic strategy against hepatocellular carcinoma. Gut.

[62] Martinez-Reyes I, Chandel NS (2021). Cancer metabolism: looking forward. Nat. Rev. Cancer.

[63] Rogers T, DeBerardinis RJ (2021). Metabolic plasticity of neutrophils: relevance to pathogen responses and cancer. Trends Cancer.

[64] Sasidharan Nair V, Saleh R, Toor SM, Cyprian FS, Elkord E (2021). Metabolic reprogramming of T regulatory cells in the hypoxic tumor microenvironment. Cancer Immunol., immunotherapy: CII.

[65] Rashed FB (2021). Identification of proteins and cellular pathways targeted by 2-nitroimidazole hypoxic cytotoxins. Redox Biol..

[66] Sengupta A, Roy SS, Chowdhury S (2021). Non-duplex G-Quadruplex DNA structure: a developing story from predicted sequences to DNA structure-dependent epigenetics and beyond. Acc. Chem. Res..

[67] Hindupur SK (2018). The protein histidine phosphatase LHPP is a tumour suppressor. Nature.

[68] Bi K (2021). Tumor and immune reprogramming during immunotherapy in advanced renal cell carcinoma. Cancer Cell.

[69] de Azevedo RA (2020). MIF inhibition as a strategy for overcoming resistance to immune checkpoint blockade therapy in melanoma. Oncoimmunology.

[70] Barkal AA (2019). CD24 signalling through macrophage Siglec-10 is a target for cancer immunotherapy. Nature.

[71] Das M, Zhu C, Kuchroo VK (2017). Tim-3 and its role in regulating anti-tumor immunity. Immunol. Rev..

[72] Lines JL (2014). VISTA is an immune checkpoint molecule for human T cells. Cancer Res..

[73] Sanmamed MF (2021). A burned-Out CD8(+) T-cell subset expands in the tumor microenvironment and curbs cancer immunotherapy. Cancer Discov..

[74] Radyk MD (2021). ATF3 induces RAB7 to govern autodegradation in paligenosis, a conserved cell plasticity program. EMBO Rep..

[75] Miao ZF, Cho CJ, Wang ZN, Mills JC (2021). Autophagy repurposes cells during paligenosis. Autophagy.

[76] Miao ZF (2021). DDIT4 licenses only healthy cells to proliferate during injury-induced metaplasia. Gastroenterology.

[77] Miao ZF (2020). A dedicated evolutionarily conserved molecular network licenses differentiated cells to return to the cell cycle. Dev. Cell.

[78] Rodgers JT (2014). mTORC1 controls the adaptive transition of quiescent stem cells from G0 to G(Alert). Nature.

[79] Goldenring JR, Mills JC (2021). Cellular plasticity, reprogramming, and regeneration: metaplasia in the stomach and beyond. Gastroenterology.

[80] Lambert AW, Weinberg RA (2021). Linking EMT programmes to normal and neoplastic epithelial stem cells. Nat. Rev. Cancer.

[81] Quintanal-Villalonga A (2020). Lineage plasticity in cancer: a shared pathway of therapeutic resistance. Nat. Rev. Clin. Oncol..

[82] Arozarena I, Wellbrock C (2019). Phenotype plasticity as enabler of melanoma progression and therapy resistance. Nat. Rev. Cancer.

[83] Yuan S, Norgard RJ, Stanger BZ (2019). Cellular plasticity in cancer. Cancer Discov..

[84] Pang MJ (2022). Gastric organoids: progress and remaining challenges. Cell. Mol. Gastroenterol. Hepatol..

[85] Seidlitz T, Koo BK, Stange DE (2021). Gastric organoids-an in vitro model system for the study of gastric development and road to personalized medicine. Cell Death Differ..

[86] Drost J, Clevers H (2018). Organoids in cancer research. Nat. Rev. Cancer.

[87] Schuijs MJ, Hammad H, Lambrecht BN (2019). Professional and ‘Amateur’ antigen-presenting cells in type 2 immunity. Trends Immunol..

[88] Gardner A, Ruffell B (2016). Dendritic cells and cancer immunity. Trends Immunol..

[89] Roche PA, Furuta K (2015). The ins and outs of MHC class II-mediated antigen processing and presentation. Nat. Rev. Immunol..

[90] Peinado H (2017). Pre-metastatic niches: organ-specific homes for metastases. Nat. Rev. Cancer.

[91] Liu Y, Cao X (2016). Characteristics and significance of the pre-metastatic niche. Cancer Cell.

[92] Bakdash G (2016). Expansion of a BDCA1+CD14+ myeloid cell population in melanoma patients may attenuate the efficacy of dendritic cell vaccines. Cancer Res..

[93] Sanchez-Paulete AR (2018). Intratumoral immunotherapy with XCL1 and sFlt3L encoded in recombinant semliki forest virus-derived vectors fosters dendritic cell-mediated T-cell cross-priming. Cancer Res..

[94] Zlotnik A, Yoshie O (2012). The chemokine superfamily revisited. Immunity.

[95] Harlin H (2009). Chemokine expression in melanoma metastases associated with CD8+ T-cell recruitment. Cancer Res..

[96] Kumar V (2021). Single-cell atlas of lineage states, tumor microenvironment and subtype-specific expression programs in gastric cancer. Cancer Discov..

[97] Cancer Genome Atlas Research, N. (2014). Comprehensive molecular characterization of gastric adenocarcinoma. Nature.

[98] Cooper J, Giancotti FG (2019). Integrin signaling in cancer: mechanotransduction, stemness, epithelial plasticity, and therapeutic resistance. Cancer Cell.

[99] Gupta PB, Pastushenko I, Skibinski A, Blanpain C, Kuperwasser C (2019). Phenotypic plasticity: driver of cancer initiation, progression, and therapy resistance. Cell Stem cell.

[100] Bayik D, Lathia JD (2021). Cancer stem cell-immune cell crosstalk in tumour progression. Nat. Rev. Cancer.

[101] Saygin C, Matei D, Majeti R, Reizes O, Lathia JD (2019). Targeting cancer stemness in the clinic: from hype to hope. Cell Stem Cell.

[102] Batlle E, Clevers H (2017). Cancer stem cells revisited. Nat. Med..

[103] Shibue T, Weinberg RA (2017). EMT, CSCs, and drug resistance: the mechanistic link and clinical implications. Nat. Rev. Clin. Oncol..

[104] DePeaux K, Delgoffe GM (2021). Metabolic barriers to cancer immunotherapy. Nat. Rev. Immunol..

[105] Thorsson V (2018). The immune landscape of cancer. Immunity.

[106] Jones CL, Inguva A, Jordan CT (2021). Targeting energy metabolism in cancer stem cells: progress and challenges in leukemia and solid tumors. Cell Stem Cell.

[107] Ferguson LP, Diaz E, Reya T (2021). The role of the microenvironment and immune system in regulating stem cell fate in cancer. Trends Cancer.

[108] Stoica AF, Chang CH, Pauklin S (2020). Molecular therapeutics of pancreatic ductal adenocarcinoma: targeted pathways and the role of cancer stem cells. Trends Pharmacol. Sci..

[109] Prager BC, Xie Q, Bao S, Rich JN (2019). Cancer stem cells: the architects of the tumor ecosystem. Cell Stem Cell.

[110] Fan QM (2014). Tumor-associated macrophages promote cancer stem cell-like properties via transforming growth factor-beta1-induced epithelial-mesenchymal transition in hepatocellular carcinoma. Cancer Lett..

[111] Jinushi M (2011). Tumor-associated macrophages regulate tumorigenicity and anticancer drug responses of cancer stem/initiating cells. Proc. Natl Acad. Sci. USA.

[112] Pietras A (2014). Osteopontin-CD44 signaling in the glioma perivascular niche enhances cancer stem cell phenotypes and promotes aggressive tumor growth. Cell Stem Cell.

[113] Williams K, Motiani K, Giridhar PV, Kasper S (2013). CD44 integrates signaling in normal stem cell, cancer stem cell and (pre)metastatic niches. Exp. Biol. Med..

[114] Zoller M (2011). CD44: can a cancer-initiating cell profit from an abundantly expressed molecule?. Nat. Rev. Cancer.

[115] Matulonis UA (2016). Ovarian cancer. Nat. Rev. Dis. Prim..

[116] Izar B (2020). A single-cell landscape of high-grade serous ovarian cancer. Nat. Med..

[117] Lordick F (2022). Gastric cancer: ESMO clinical practice guideline for diagnosis, treatment and follow-up. Ann. Oncol..

[118] Pimentel-Nunes P (2022). Endoscopic submucosal dissection for superficial gastrointestinal lesions: European Society of Gastrointestinal Endoscopy (ESGE) Guideline - Update 2022. Endoscopy.

[119] Ono H (2021). Guidelines for endoscopic submucosal dissection and endoscopic mucosal resection for early gastric cancer (second edition). Dig. Endosc..

[120] Van Cutsem E, Sagaert X, Topal B, Haustermans K, Prenen H (2016). Gastric cancer. Lancet.

[121] Committee, A.S.O.P. (2015). The role of endoscopy in the management of premalignant and malignant conditions of the stomach. Gastrointest. Endosc..

[122] Japanese Gastric Cancer, A. (2011). Japanese classification of gastric carcinoma: 3rd English edition. Gastric Cancer.

[123] McGinnis CS, Murrow LM, Gartner ZJ (2019). DoubletFinder: doublet detection in single-cell RNA sequencing data using artificial nearest neighbors. Cell Syst..

[124] Stuart T (2019). Comprehensive integration of single-cell data. Cell.

[125] Korsunsky I (2019). Fast, sensitive and accurate integration of single-cell data with Harmony. Nat. Methods.

[126] Ren X (2021). COVID-19 immune features revealed by a large-scale single-cell transcriptome atlas. Cell.

[127] Palovics R (2022). Molecular hallmarks of heterochronic parabiosis at single-cell resolution. Nature.

[128] Au L (2021). Determinants of anti-PD-1 response and resistance in clear cell renal cell carcinoma. Cancer Cell.

[129] Aran D (2019). Reference-based analysis of lung single-cell sequencing reveals a transitional profibrotic macrophage. Nat. Immunol..

[130] Venteicher AS (2017). Decoupling genetics, lineages, and microenvironment in IDH-mutant gliomas by single-cell RNA-seq. Science.

[131] Patel AP (2014). Single-cell RNA-seq highlights intratumoral heterogeneity in primary glioblastoma. Science.

[132] Qiu X (2017). Reversed graph embedding resolves complex single-cell trajectories. Nat. Methods.

[133] Qiu X (2017). Single-cell mRNA quantification and differential analysis with census. Nat. Methods.

[134] Zhang L (2018). Lineage tracking reveals dynamic relationships of T cells in colorectal cancer. Nature.

[135] Guo X (2018). Global characterization of T cells in non-small-cell lung cancer by single-cell sequencing. Nat. Med..

[136] Gaude E, Frezza C (2016). Tissue-specific and convergent metabolic transformation of cancer correlates with metastatic potential and patient survival. Nat. Commun..

[137] Belinky F (2015). PathCards: multi-source consolidation of human biological pathways. Database.

[138] Hanzelmann S, Castelo R, Guinney J (2013). GSVA: gene set variation analysis for microarray and RNA-seq data. BMC Bioinforma..

[139] Subramanian A (2005). Gene set enrichment analysis: a knowledge-based approach for interpreting genome-wide expression profiles. Proc. Natl Acad. Sci. USA.

[140] Jassal B (2020). The reactome pathway knowledgebase. Nucleic Acids Res..

[141] Liberzon A (2015). The Molecular Signatures Database (MSigDB) hallmark gene set collection. Cell Syst..

[142] Yu G, Wang LG, Han Y, He QY (2012). clusterProfiler: an R package for comparing biological themes among gene clusters. Omics.

[143] Ashburner M (2000). Gene ontology: tool for the unification of biology. The gene ontology consortium. Nat. Genet..

[144] Efremova M, Vento-Tormo M, Teichmann SA, Vento-Tormo R (2020). CellPhoneDB: inferring cell-cell communication from combined expression of multi-subunit ligand-receptor complexes. Nat. Protoc..

[145] Aibar S (2017). SCENIC: single-cell regulatory network inference and clustering. Nat. Methods.

