# Supplementary material for: Single-cell sequencing of ascites fluid illustrates heterogeneity and therapy-induced evolution during gastric cancer peritoneal metastasis
Source: Nat Commun. 2023 Feb 14;14:822. doi: 10.1038/s41467-023-36310-9 (PMC9929081; doi:10.1038/s41467-023-36310-9)
Supplement: Supplementary file 1 — Supplementary Info [file 41467_2023_36310_MOESM1_ESM.pdf]

Single-cell sequencing of ascites fluid illustrates heterogeneity and therapy-induced evolution during gastric cancer peritoneal metastasis

Supplementary Figure and Supplementary Tables:

Supplementary Figure and Supplementary Figure Legends:

Supplementary Fig. 1-7

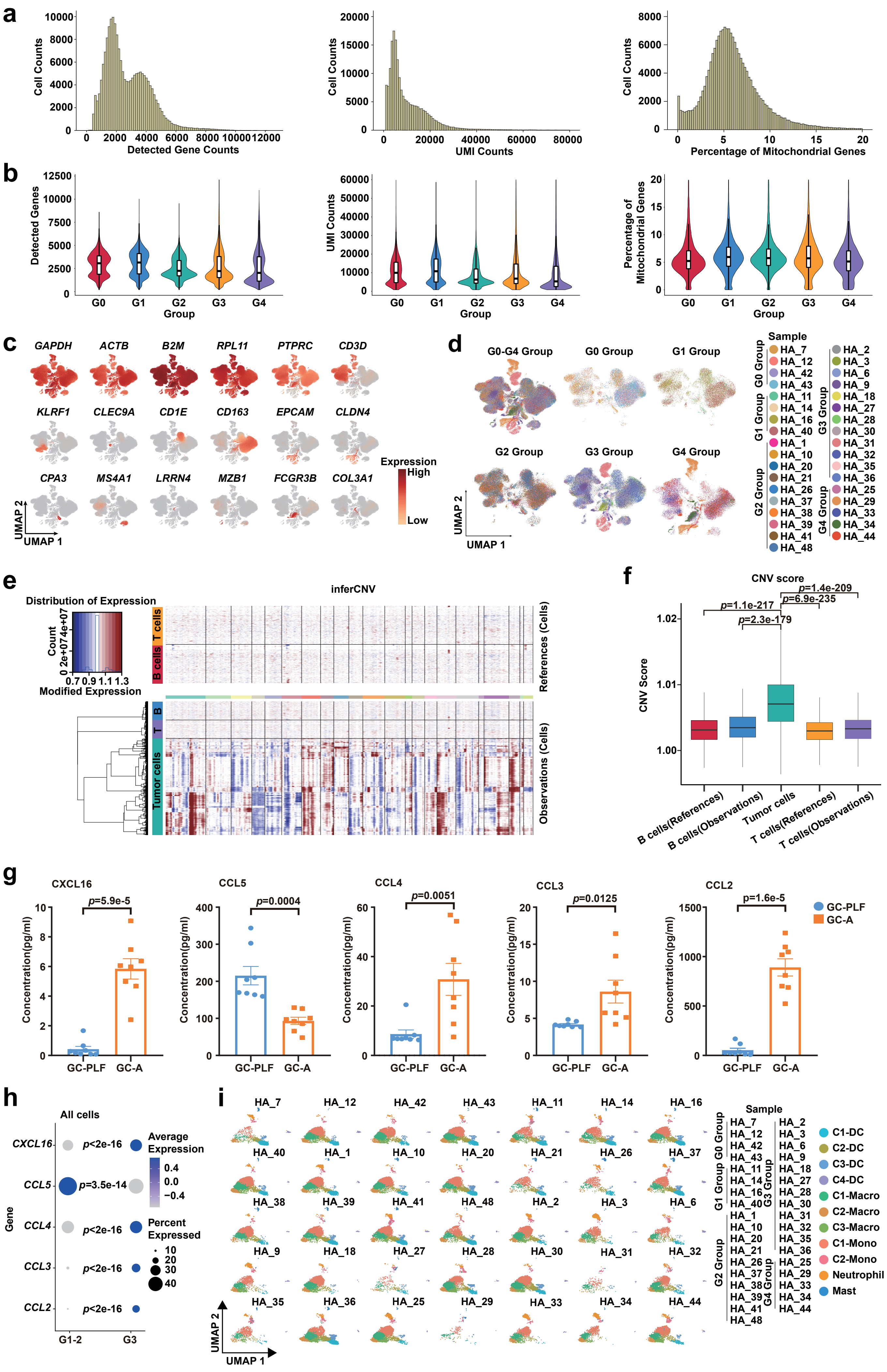
a
Cell Counts
Detected Gene Counts
UMI Counts
Percentage of Mitochondrial Genes
b
Detected Genes
UMI Counts
Percentage of Mitochondrial Genes
G0 G1 G2 G3 G4
Group
c
GAPDH ACTB B2M RPL11 PTPRC CD3D
KLRF1 CLEC9A CD1E CD163 EPCAM CLDN4
CPA3 MS4A1 LRRN4 MZB1 FCGR3B COL3A1
Expression
High
Low
UMAP 2
UMAP 1
d
G0-G4 Group
G0 Group
G1 Group
G2 Group
G3 Group
G4 Group
Sample
HA_7 HA_12 HA_42 HA_43 HA_11 HA_14 HA_16 HA_40 HA_1 HA_10 HA_20 HA_21 HA_26 HA_37 HA_38 HA_39 HA_41 HA_48
HA_2 HA_3 HA_6 HA_9 HA_18 HA_27 HA_28 HA_30 HA_31 HA_32 HA_35 HA_36 HA_25 HA_29 HA_33 HA_34 HA_44
e
inferCNV
Distribution of Expression
Count
Modified Expression
T cells
B cells
References (Cells)
B
T
Tumor cells
Observations (Cells)
f
CNV score
p=1.4e-209
p=1.1e-217
p=6.9e-235
p=2.3e-179
CNV Score
B cells(References)
B cells(Observations)
Tumor cells
T cells(References)
T cells(Observations)
g
CXCL16 p=5.9e-5
CCL5 p=0.0004
CCL4 p=0.0051
CCL3 p=0.0125
CCL2 p=1.6e-5
Concentration(pg/ml)
GC-PLF GC-A
h
All cells
CXCL16 p<2e-16
CCL5 p=3.5e-14
CCL4 p<2e-16
CCL3 p<2e-16
CCL2 p<2e-16
Gene
G1-2 G3
Average Expression
Percent Expressed
i
Sample
C1-DC C2-DC C3-DC C4-DC C1-Macro C2-Macro C3-Macro C1-Mono C2-Mono Neutrophil Mast
UMAP 2
UMAP 1

Supplementary Fig. 1. scRNA-seq profiles of cells identified in the peritoneal ecosystem

(a) Histogram showing the distributions of detected gene counts, unique molecular identifier (UMI) counts, and percentage of mitochondrial genes per cell.

(b) Violin plots showing the distributions of detected genes, UMI counts and percentage of mitochondrial genes for cells in G0-G4 Groups (G0: n = 19581 cells; G1: n = 16768 cells; G2: n = 56153 cells; G3: n = 69648 cells; G4: n = 29837 cells). Box represents median ± interquartile range; whiskers represent 1.5x interquartile range.

(c) Uniform Manifold Approximation and Projection (UMAP) plots indicating the expression of marker genes to annotate cell types.

(d) Uniform Manifold Approximation and Projection (UMAP) plot showing the cell type distribution in G0-G4 Groups. Each color represents a sample.

(e) Heatmap indicating chromosomal copy number variation (CNV) of T cells, B cells, and tumor cells. Upper panel shows the CNVs of 1000 random-selected T and B cells as reference cells. Lower panel shows the CNVs of another 1000 random-selected T and B cells for validation and all tumor cells for observation.

(f) Box plots showing CNV scores of T cells (n = 1000 cells) and B cells (n = 1000 cells) in references and observations, respectively, and tumor cells (n = 5235 cells). Box represents median ± interquartile range; whiskers represent 1.5x interquartile range; the *p* values are calculated by two-sided unpaired Wilcoxon test.

(g) ELISA of chemokines in the gastric cancer ascites (GC-A, n = 8 samples) and gastric cancer peritoneal lavage fluids (GC-PLF, n = 8 samples). Data are presented as mean ± SEM; the *p* values are calculated by two-sided unpaired Student's t test.

(h) Dotplots showing the expression levels of chemokine genes in all cells. Dot size represents percent of expressing cells in each group, color represents expression level of selected genes. G1-2: gastric cancer without peritoneal metastasis, as GC-PLF in (g); G3, gastric cancer with peritoneal metastasis, as GC-A in (g). *p* values are calculated by Wilcoxon test.

(i) UMAP plot showing the distribution of myeloid cells in G0-G4 Groups. Each color represents a cell type.

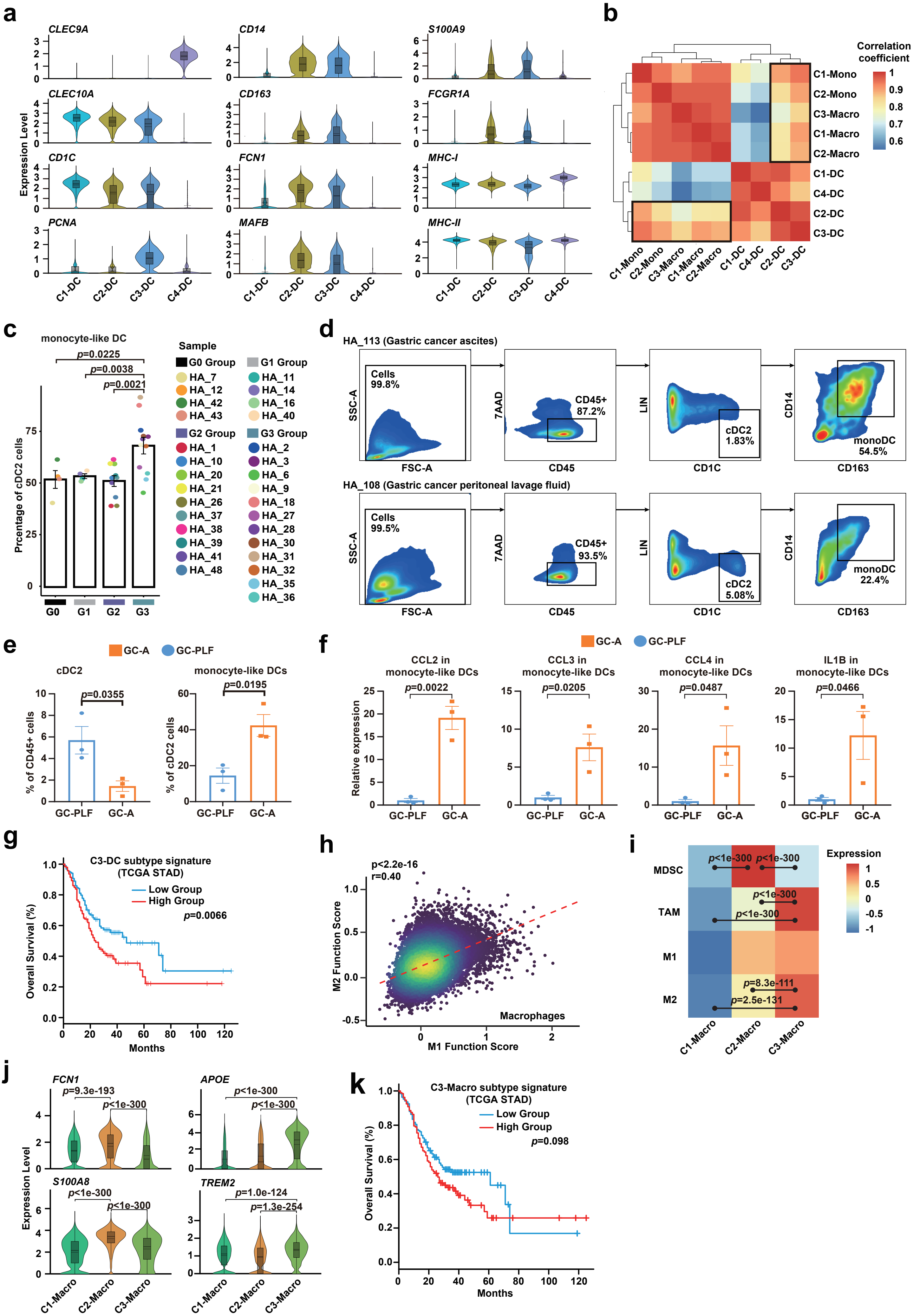

a
CLEC9A
CD14
S100A9
CLEC10A
CD163
FCGR1A
Expression Level
CD1C
FCN1
MHC-I
PCNA
MAFB
MHC-II
C1-DC
C2-DC
C3-DC
C4-DC
b
Correlation coefficient
C1-Mono
C2-Mono
C3-Macro
C1-Macro
C2-Macro
C1-DC
C4-DC
C2-DC
C3-DC
c
monocyte-like DC
p=0.0225
p=0.0038
p=0.0021
Prcentage of cDC2 cells
Sample
G0 Group
G1 Group
G2 Group
G3 Group
G0
G1
G2
G3
d
HA_113 (Gastric cancer ascites)
HA_108 (Gastric cancer peritoneal lavage fluid)
Cells 99.8%
CD45+ 87.2%
cDC2 1.83%
monoDC 54.5%
Cells 99.5%
CD45+ 93.5%
cDC2 5.08%
monoDC 22.4%
SSC-A
FSC-A
7AAD
CD45
LIN
CD1C
CD14
CD163
e
GC-A
GC-PLF
cDC2
p=0.0355
% of CD45+ cells
monocyte-like DCs
p=0.0195
% of cDC2 cells
f
CCL2 in monocyte-like DCs
p=0.0022
CCL3 in monocyte-like DCs
p=0.0205
CCL4 in monocyte-like DCs
p=0.0487
IL1B in monocyte-like DCs
p=0.0466
Relative expression
g
C3-DC subtype signature (TCGA STAD)
Low Group
High Group
p=0.0066
Overall Survival (%)
Months
h
p<2.2e-16
r=0.40
M2 Function Score
M1 Function Score
Macrophages
i
Expression
MDSC
TAM
M1
M2
p<1e-300
p=8.3e-111
p=2.5e-131
C1-Macro
C2-Macro
C3-Macro
j
FCN1
p=9.3e-193
p<1e-300
APOE
S100A8
TREM2
p=1.0e-124
p=1.3e-254
Expression Level
k
C3-Macro subtype signature (TCGA STAD)
p=0.098

Supplementary Fig. 2. Characteristics of myeloid cells in the peritoneal microenvironment during gastric cancer peritoneal metastasis

(a) Violin plots showing the expression levels of selected marker genes in dendritic cell (DC) (C1-DC: n = 8753 cells; C2-DC: n = 8430 cells; C3-DC: n = 2173 cells; C4-DC: n = 921 cells), color-coded by cell type. Box represents median ± interquartile range; whiskers represent 1.5x interquartile range; horizontal dotted line represents mean value.

(b) Heatmap showing the correlation between myeloid subtypes. Black boxes highlight the C2/C3-DC sharing with monocyte-lineage features.

(c) The proportion of monocyte-like DCs in different groups from G0 (n = 4 samples), G1 (n = 4 samples), G2 (n = 10 samples) and G3 (n = 12 samples) Group. Each color represents a sample. Data are presented as mean values ± SEM (error bars); the *p* values are calculated by two-sided unpaired Student's t test.

(d) FACS strategy of monocyte-like DCs from gastric cancer ascites and gastric cancer peritoneal lavage fluids.

(e) Bar plots showing the proportions of cDC2 in CD45+ cells (Left) and monocyte-like DCs in cDC2 (Right) between gastric cancer ascites (GC-A, n= 3 independent experiments) and gastric cancer peritoneal lavage fluids (GC-PLF, n= 3 independent experiments). Data are presented as mean values ± SEM (error bars); the *p* values are calculated by two-sided unpaired Student's t test.

(f) Bar plots showing qPCR results on chemokines for monocyte-like DCs between gastric cancer ascites (GC-A, n= 3 independent experiments) and gastric cancer peritoneal lavage fluids (GC-PLF, n= 3 independent experiments). Data are presented as mean values ± SEM (error bars); the *p* values are calculated by two-sided unpaired Student's t test.

(g) Kaplan-Meier curve showing the overall survival of patients from The Cancer Genome Atlas Stomach Adenocarcinoma (TCGA STAD) database based on the expression level of the C3-DC signature. *p* value is calculated by log-rank test.

(h) Scatter plots showing the correlation between M1 and M2 function score in macrophages. *p* value is calculated by Pearson correlation test.

(i) Heatmap indicating the expression levels of specific gene signatures in macrophage subtypes. Macro, macrophage. *p* values are calculated by Wilcoxon test.

(j) Violin plots indicating the expression levels of *FCN1, APOE, S100A8* and *TREM2* genes in macrophage types (C1-Macro: n = 16571 cells; C2-Macro: n = 9995 cells; C3-Macro: n = 6323 cells). Box represents median ± interquartile range; whiskers represent 1.5x interquartile range; horizontal dotted line represents mean value. The *p* values are calculated by Wilcoxon test.

(k) The overall survival of TCGA STAD patients with high or low expression group of the C3-Macro subtype signature. *p* value is calculated by log-rank test.

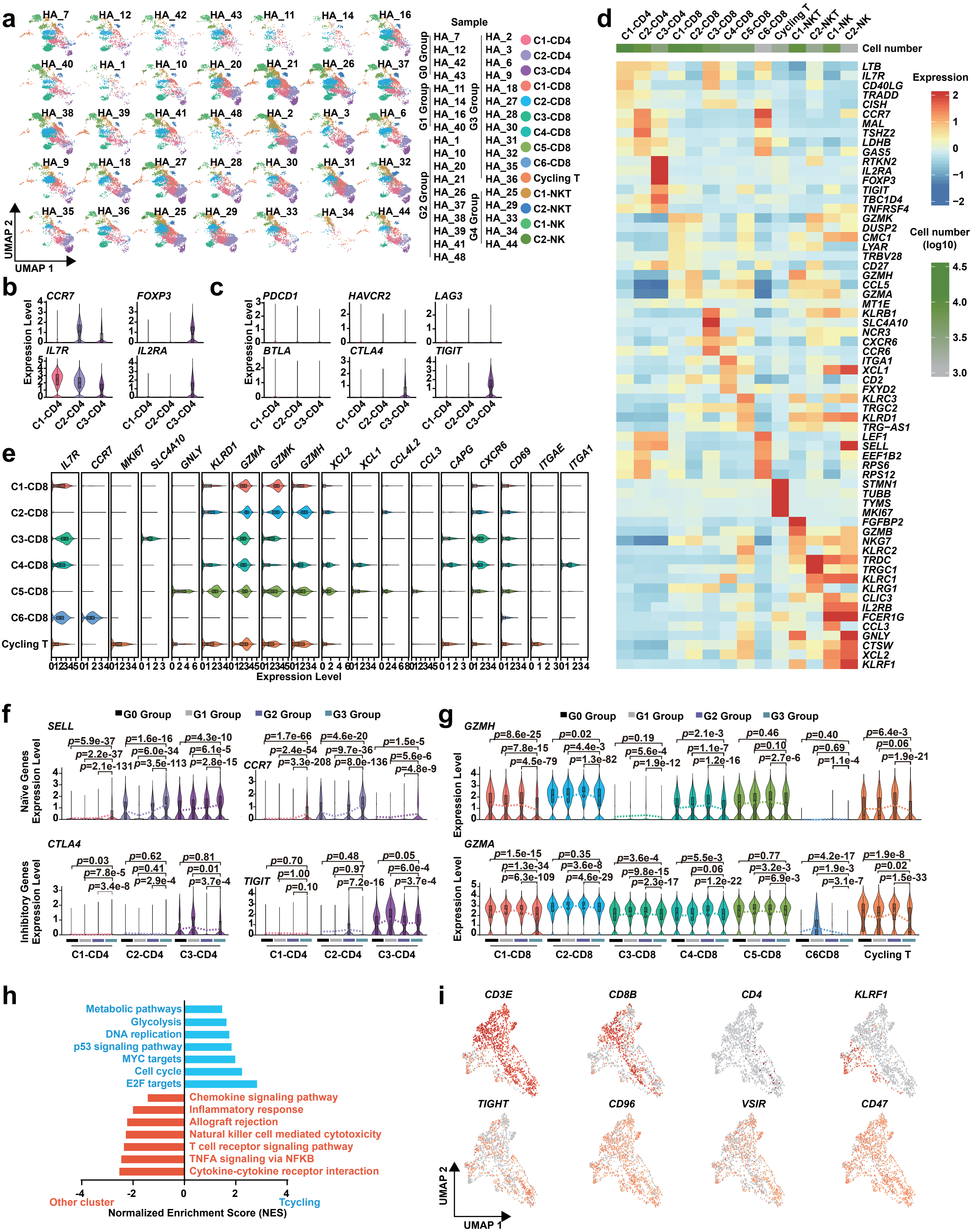

a
HA_7 HA_12 HA_42 HA_43 HA_11 HA_14 HA_16
HA_40 HA_1 HA_10 HA_20 HA_21 HA_26 HA_37
HA_38 HA_39 HA_41 HA_48 HA_2 HA_3 HA_6
HA_9 HA_18 HA_27 HA_28 HA_30 HA_31 HA_32
HA_35 HA_36 HA_25 HA_29 HA_33 HA_34 HA_44
UMAP 2
UMAP 1
Sample
G0 Group
G1 Group
G2 Group
G3 Group
G4 Group
C1-CD4 C2-CD4 C3-CD4 C1-CD8 C2-CD8 C3-CD8 C4-CD8 C5-CD8 C6-CD8 Cycling T C1-NKT C2-NKT C1-NK C2-NK
b
CCR7 FOXP3 IL7R IL2RA
Expression Level
C1-CD4 C2-CD4 C3-CD4
c
PDCD1 HAVCR2 LAG3 BTLA CTLA4 TIGIT
Expression Level
d
Cell number
Expression
Cell number (log10)
LTB IL7R CD40LG TRADD CISH CCR7 MAL TSHZ2 LDHB GAS5 RTKN2 IL2RA FOXP3 TIGIT TBC1D4 TNFRSF4 GZMK DUSP2 CMC1 LYAR TRBV28 CD27 GZMH CCL5 GZMA MT1E KLRB1 SLC4A10 NCR3 CXCR6 CCR6 ITGA1 XCL1 CD2 FXYD2 KLRC3 TRGC2 KLRD1 TRG-AS1 LEF1 SELL EEF1B2 RPS6 RPS12 STMN1 TUBB TYMS MKI67 FGFBP2 GZMB NKG7 KLRC2 TRDC TRGC1 KLRC1 KLRG1 CLIC3 IL2RB FCER1G CCL3 GNLY CTSW XCL2 KLRF1
e
IL7R CCR7 MKI67 SLC4A10 GNLY KLRD1 GZMA GZMK GZMH XCL2 XCL1 CCL4L2 CCL3 CAPG CXCR6 CD69 ITGAE ITGA1
C1-CD8 C2-CD8 C3-CD8 C4-CD8 C5-CD8 C6-CD8 Cycling T
Expression Level
f
G0 Group G1 Group G2 Group G3 Group
SELL CCR7
Naïve Genes Expression Level
CTLA4 TIGIT
Inhibitory Genes Expression Level
C1-CD4 C2-CD4 C3-CD4
g
G0 Group G1 Group G2 Group G3 Group
GZMH GZMA
Expression Level
C1-CD8 C2-CD8 C3-CD8 C4-CD8 C5-CD8 C6CD8 Cycling T
h
Metabolic pathways
Glycolysis
DNA replication
p53 signaling pathway
MYC targets
Cell cycle
E2F targets
Chemokine signaling pathway
Inflammatory response
Allograft rejection
Natural killer cell mediated cytotoxicity
T cell receptor signaling pathway
TNFA signaling via NFKB
Cytokine-cytokine receptor interaction
Other cluster
Tcycling
Normalized Enrichment Score (NES)
i
CD3E CD8B CD4 KLRF1
TIGHT CD96 VSIR CD47
UMAP 2
UMAP 1

Supplementary Fig. 3. Characteristics of T cells in the peritoneal microenvironment during gastric cancer peritoneal metastasis

(a) Uniform Manifold Approximation and Projection (UMAP) plot showing the distribution of T/NK cells in different groups. Each color represents a cell type.

(b, c) Violin plots showing expression levels of marker genes (b) and inhibitory genes (c) in CD4 subtypes (C1-CD4: n = 12855 cells; C2-CD4: n = 8528 cells; C3-CD4: n = 3485 cells). Box represents median ± interquartile range; whiskers represent 1.5x interquartile range; horizontal dotted line represents mean value.

(d) Heatmap plot indicating expression levels of selected genes among T/NK cell types. Expression level is projected by color: red represents high expression and blue represents low expression.

(e) Violin plots of selected marker genes (columns) for CD8 T cell subsets and cycling T cells (rows) (C1-CD8: n = 11021 cells; C2-CD8: n = 10285 cells; C3-CD8: n = 5623 cells; C4-CD8: n = 4025 cells; C5-CD8: n = 4007 cells; Cycling T: n = 1404 cells).

(f) Violin plot showing expression levels of selected naïve and inhibitory genes in CD4 T cells in G0 (n = 4 samples), G1 (n = 4 samples), G2 (n = 10 samples) and G3 (n = 12 samples) Group. Horizontal dotted line represents mean value. Box represents median ± interquartile range; whiskers represent 1.5x interquartile range; the *p* values are calculated by two-sided unpaired Wilcoxon test.

(g) Violin plot showing expression levels of selected cytotoxic genes in CD8 T cell types and cycling T cells in G0 (n = 4 samples), G1 (n = 4 samples), G2 (n = 10 samples) and G3 (n = 12 samples) Group. Horizontal dotted line represents mean value. Box represents median ± interquartile range; whiskers represent 1.5x interquartile range; the *p* values are calculated by two-sided unpaired Wilcoxon test.

(h) Bar chart showing the normalized enrichment score (NES) of specific pathways enriched in cycling T (blue lines) and other CD8 T cells (red lines) based on Gene Set Enrichment Analyses (GSEA) analysis.

(i) Uniform Manifold Approximation and Projection (UMAP) plots indicating the expression of selected marker genes to determine cycling T subtypes.

a

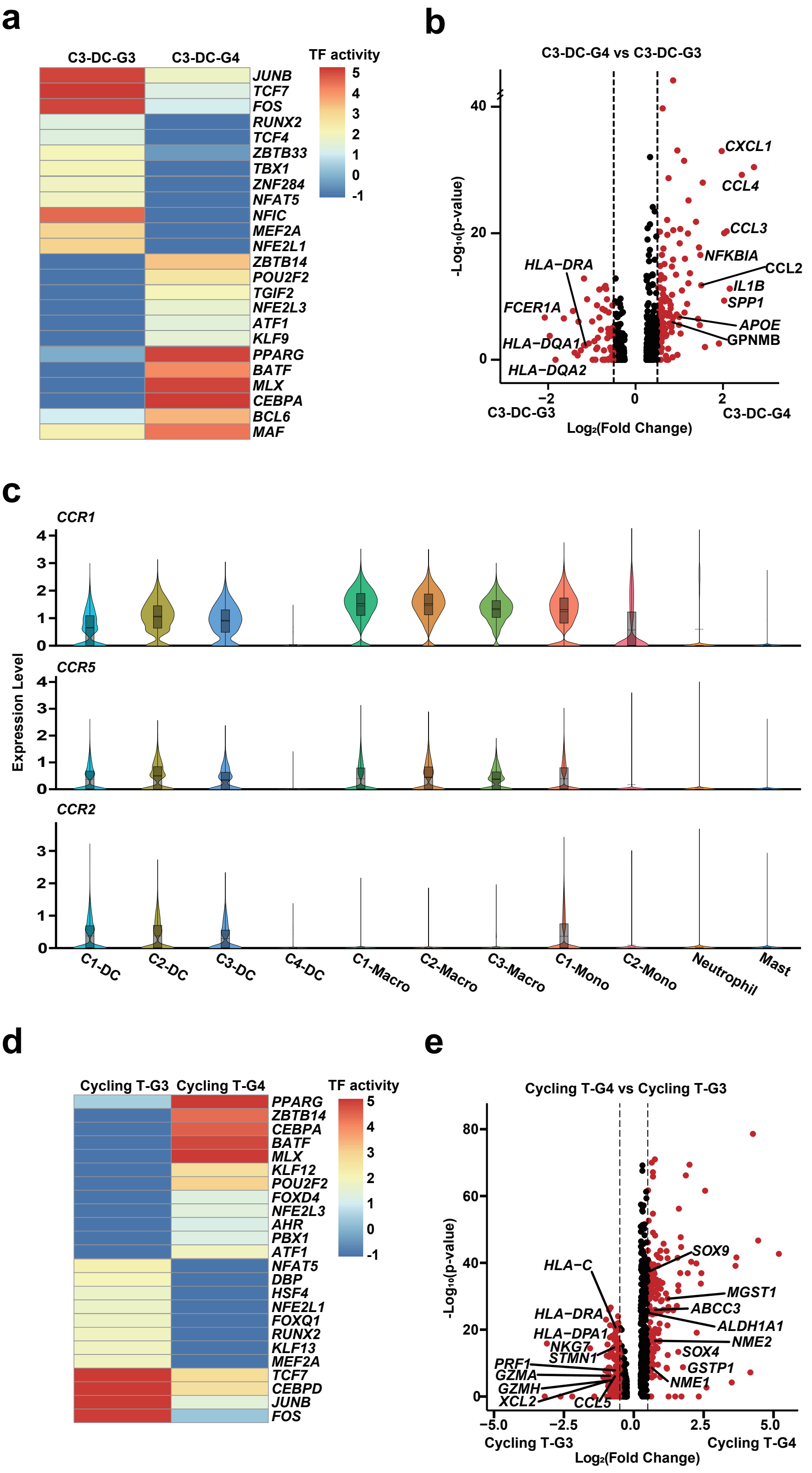

C3-DC-G3
C3-DC-G4
TF activity
JUNB
TCF7
FOS
RUNX2
TCF4
ZBTB33
TBX1
ZNF284
NFAT5
NFIC
MEF2A
NFE2L1
ZBTB14
POU2F2
TGIF2
NFE2L3
ATF1
KLF9
PPARG
BATF
MLX
CEBPA
BCL6
MAF
b
C3-DC-G4 vs C3-DC-G3
-Log10(p-value)
Log2(Fold Change)
C3-DC-G3
C3-DC-G4
CXCL1
CCL4
CCL3
NFKBIA
CCL2
IL1B
SPP1
APOE
GPNMB
HLA−DRA
FCER1A
HLA−DQA1
HLA−DQA2
c
CCR1
CCR5
CCR2
Expression Level
C1-DC
C2-DC
C3-DC
C4-DC
C1-Macro
C2-Macro
C3-Macro
C1-Mono
C2-Mono
Neutrophil
Mast
d
Cycling T-G3
Cycling T-G4
TF activity
PPARG
ZBTB14
CEBPA
BATF
MLX
KLF12
POU2F2
FOXD4
NFE2L3
AHR
PBX1
ATF1
NFAT5
DBP
HSF4
NFE2L1
FOXQ1
RUNX2
KLF13
MEF2A
TCF7
CEBPD
JUNB
FOS
e
Cycling T-G4 vs Cycling T-G3
-Log10(p-value)
Log2(Fold Change)
Cycling T-G3
Cycling T-G4
SOX9
MGST1
ABCC3
ALDH1A1
NME2
SOX4
GSTP1
NME1
HLA−C
HLA−DRA
HLA−DPA1
NKG7
STMN1
PRF1
GZMA
GZMH
XCL2
CCL5

Supplementary Fig. 4. Distinct states of monocyte-like DC and cycling T cells in therapy-naïve and therapy groups

(a) Heatmap showing transcription factor (TF) activity of C3-DC in the G3 and G4 Groups using SCENIC analysis.

(b) Volcano plot showing differentially expressed genes between C3-DC in the G3 and G4 Groups.

(c) Violin plots indicating expression levels of chemokine receptors in myeloid cells (C1-DC: n = 8753 cells; C2-DC: n = 8430 cells; C3-DC: n = 2173 cells; C4-DC: n = 921 cells; C1-Macro: n = 16571 cells; C2-Macro: n = 9995 cells; C3-Macro: n = 6323 cells; C1-Mono: n = 35729 cells; C2-Mono: n = 3682 cells; Neutrophil: n = 6602 cells; Mast: n = 1504 cells). Box represents median ± interquartile range; whiskers represent 1.5x interquartile range; horizontal dotted line represents mean value. DC, dendritic cell; Macro, macrophage; Mono, monocyte.

(d) Heatmap showing TF activity of cycling T cells in the G3 (n = 531 cells) and G4 (n = 137 cells) Groups using SCENIC analysis.

(e) Volcano plot showing differentially expressed genes between cycling T cells in the G3 and G4 Groups.

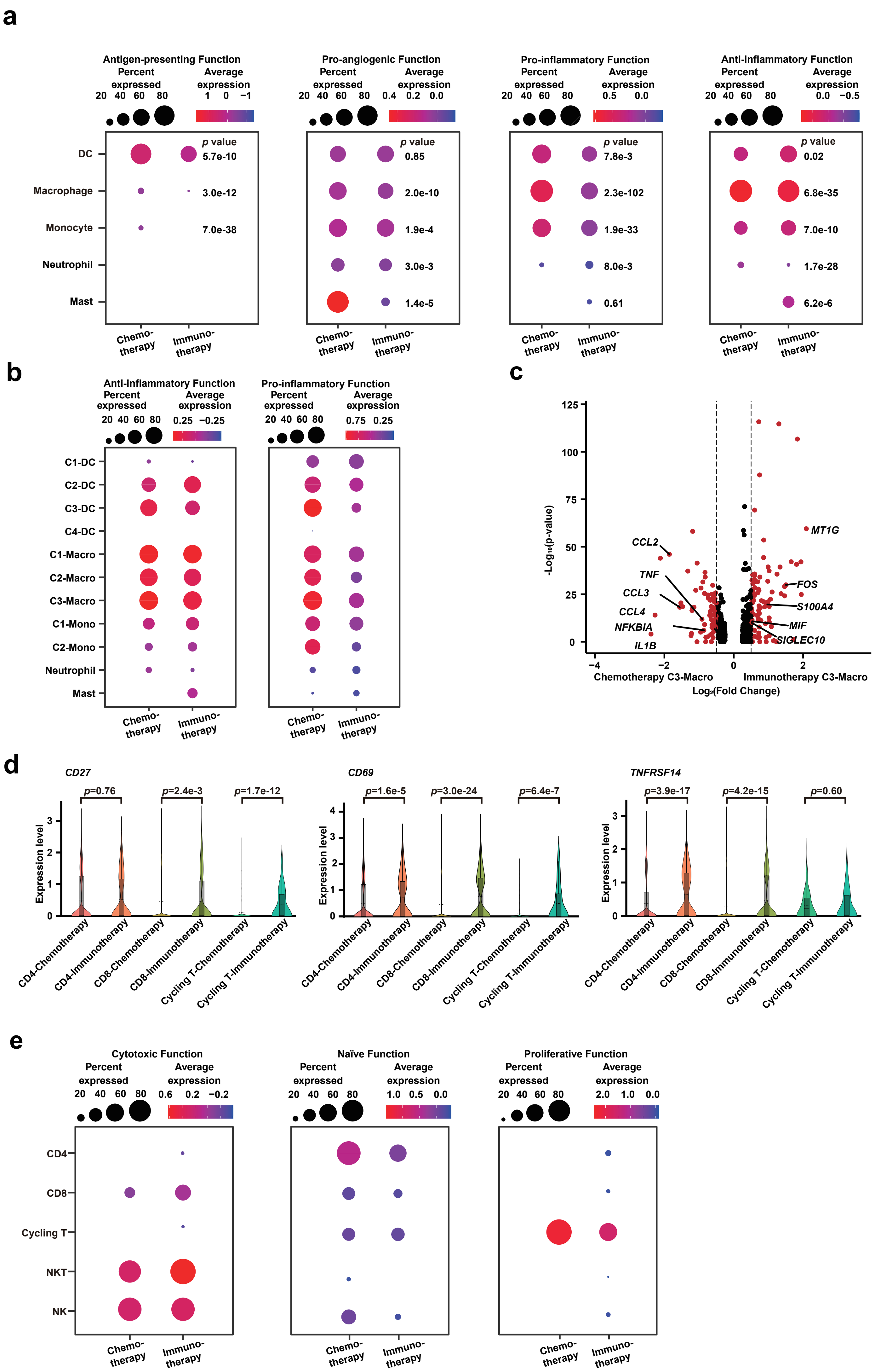
a
Antigen-presenting Function
Pro-angiogenic Function
Pro-inflammatory Function
Anti-inflammatory Function
Percent expressed
Average expression
p value
DC
Macrophage
Monocyte
Neutrophil
Mast
5.7e-10
3.0e-12
7.0e-38
0.85
2.0e-10
1.9e-4
3.0e-3
1.4e-5
7.8e-3
2.3e-102
1.9e-33
8.0e-3
0.61
0.02
6.8e-35
7.0e-10
1.7e-28
6.2e-6
Chemo-therapy
Immuno-therapy
b
Anti-inflammatory Function
Pro-inflammatory Function
C1-DC
C2-DC
C3-DC
C4-DC
C1-Macro
C2-Macro
C3-Macro
C1-Mono
C2-Mono
Neutrophil
Mast
c
-Log10(p-value)
Log2(Fold Change)
Chemotherapy C3-Macro
Immunotherapy C3-Macro
CCL2
TNF
CCL3
CCL4
NFKBIA
IL1B
MT1G
FOS
S100A4
MIF
SIGLEC10
d
CD27
CD69
TNFRSF14
p=0.76
p=2.4e-3
p=1.7e-12
p=1.6e-5
p=3.0e-24
p=6.4e-7
p=3.9e-17
p=4.2e-15
p=0.60
Expression level
CD4-Chemotherapy
CD4-Immunotherapy
CD8-Chemotherapy
CD8-Immunotherapy
Cycling T-Chemotherapy
Cycling T-Immunotherapy
e
Cytotoxic Function
Naïve Function
Proliferative Function
CD4
CD8
Cycling T
NKT
NK

Supplementary Fig. 5. Immune phenotype evolution of monocyte-like DC and cycling T cells after immunotherapy

(a) Dotplots showing the cell expression percentage (dot size) and related function scores (dot color) of antigen-presenting, pro-angiogenic, pro-inflammatory, and anti-inflammatory function scores in main myeloid subtypes in the Chemotherapy (three patients) and Immunotherapy (two patients) groups. DC, dendritic cell. *p* values are calculated by two-sided unpaired Wilcoxon test.

(b) Similar to (a), dotplots showing anti-inflammatory and pro-inflammatory function scores of myeloid subtypes in Chemotherapy (three patients) and Immunotherapy (two patients) groups. DC, dendritic cell; Macro, macrophage; Mono, monocyte.

(c) Volcano plot showing differentially expressed genes of C3-Macro cells between the Chemotherapy (three patients) and Immunotherapy (two patients) groups.

(d) Violin plot showing expression levels of co-stimulatory markers of T cells in the Chemotherapy (three patients) and Immunotherapy (two patients) groups. Box represents median ± interquartile range; whiskers represent 1.5x interquartile range; horizontal dotted line represents mean value. *p* values are calculated by two-sided unpaired Wilcoxon test.

(e) Similar to (a), dotplots showing the cytotoxic, naïve, and proliferative function scores of main T/NK cell clusters in the Chemotherapy (three patients) and Immunotherapy (two patients) groups.

Source data are provided as a Source Data file.

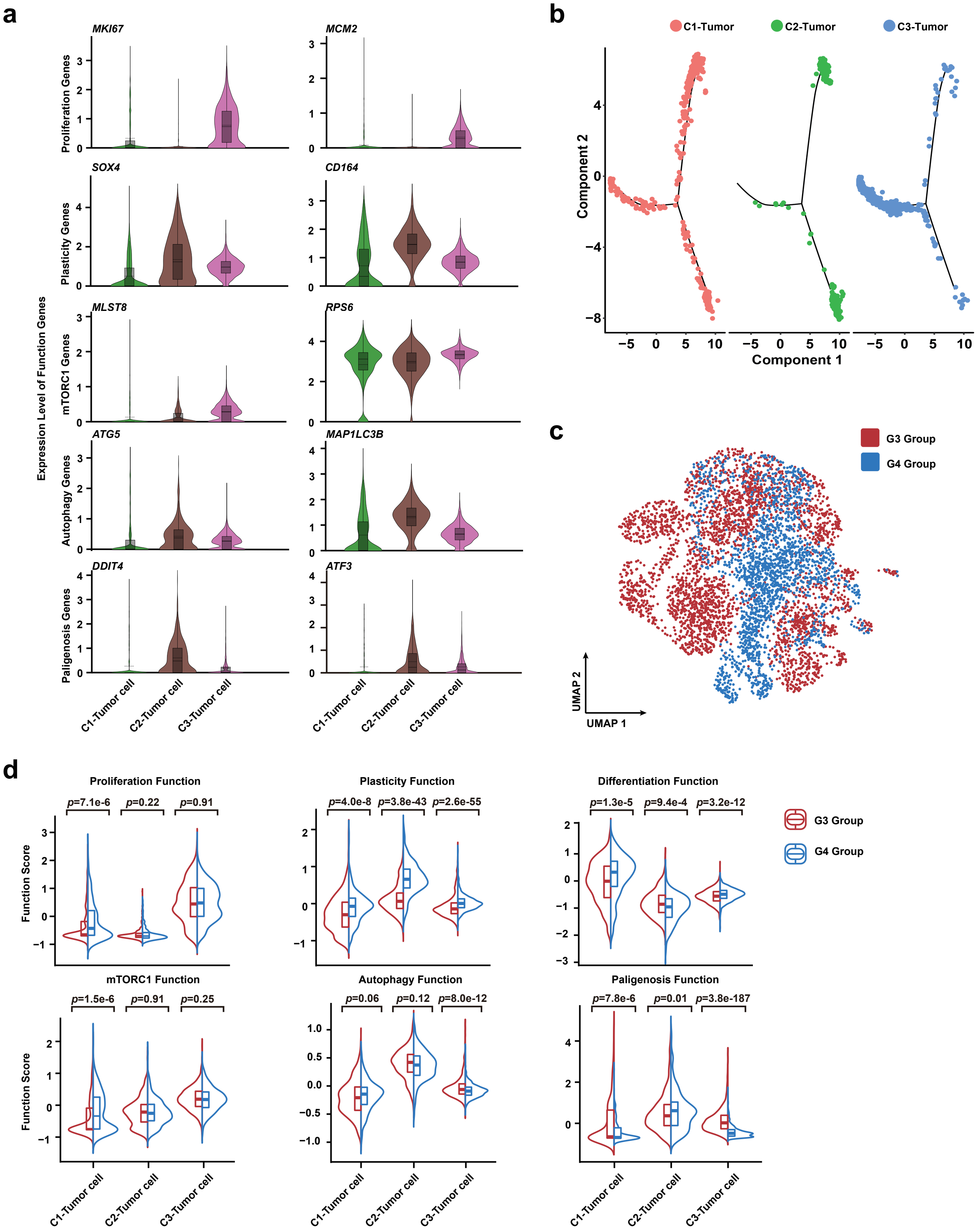

a
b
c
d
Expression Level of Function Genes
Proliferation Genes
Plasticity Genes
mTORC1 Genes
Autophagy Genes
Paligenosis Genes
MKI67
MCM2
SOX4
CD164
MLST8
RPS6
ATG5
MAP1LC3B
DDIT4
ATF3
C1-Tumor cell
C2-Tumor cell
C3-Tumor cell
C1-Tumor
C2-Tumor
C3-Tumor
Component 2
Component 1
G3 Group
G4 Group
UMAP 2
UMAP 1
Proliferation Function
p=7.1e-6 p=0.22 p=0.91
Plasticity Function
p=4.0e-8 p=3.8e-43 p=2.6e-55
Differentiation Function
p=1.3e-5 p=9.4e-4 p=3.2e-12
mTORC1 Function
p=1.5e-6 p=0.91 p=0.25
Autophagy Function
p=0.06 p=0.12 p=8.0e-12
Paligenosis Function
p=7.8e-6 p=0.01 p=3.8e-187
Function Score

Supplementary Fig. 6. Characteristics of tumor cells in the peritoneal microenvironment during gastric cancer peritoneal metastasis

(a) Violin plots showing expression levels of function-related genes, including proliferation, plasticity, mTORC1, autophagy, and paligenosis genes, in tumor subtypes (C1-Tumor cell: n = 713 cells; C2-Tumor cell: n = 549 cells; C3-Tumor cell: n = 2545 cells). Box represents median ± interquartile range; whiskers represent 1.5x interquartile range; horizontal dotted line represents mean value.

(b) Transition trajectories of tumor cell subtypes along pseudotime. Each dot corresponds to a single cell.

(c) Uniform Manifold Approximation and Projection (UMAP) plot showing the distribution of tumor cells in G3 (Red) and G4 (blue) Groups.

(d) Split violin plots representing function scores of proliferation, plasticity, differentiation, mTORC1, autophagy, and paligenosis for tumor cell subtypes in the G3 (red) and G4 (blue) Groups. Box represents median ± interquartile range; *p* values are calculated by Wilcoxon test.

Source data are provided as a Source Data file.

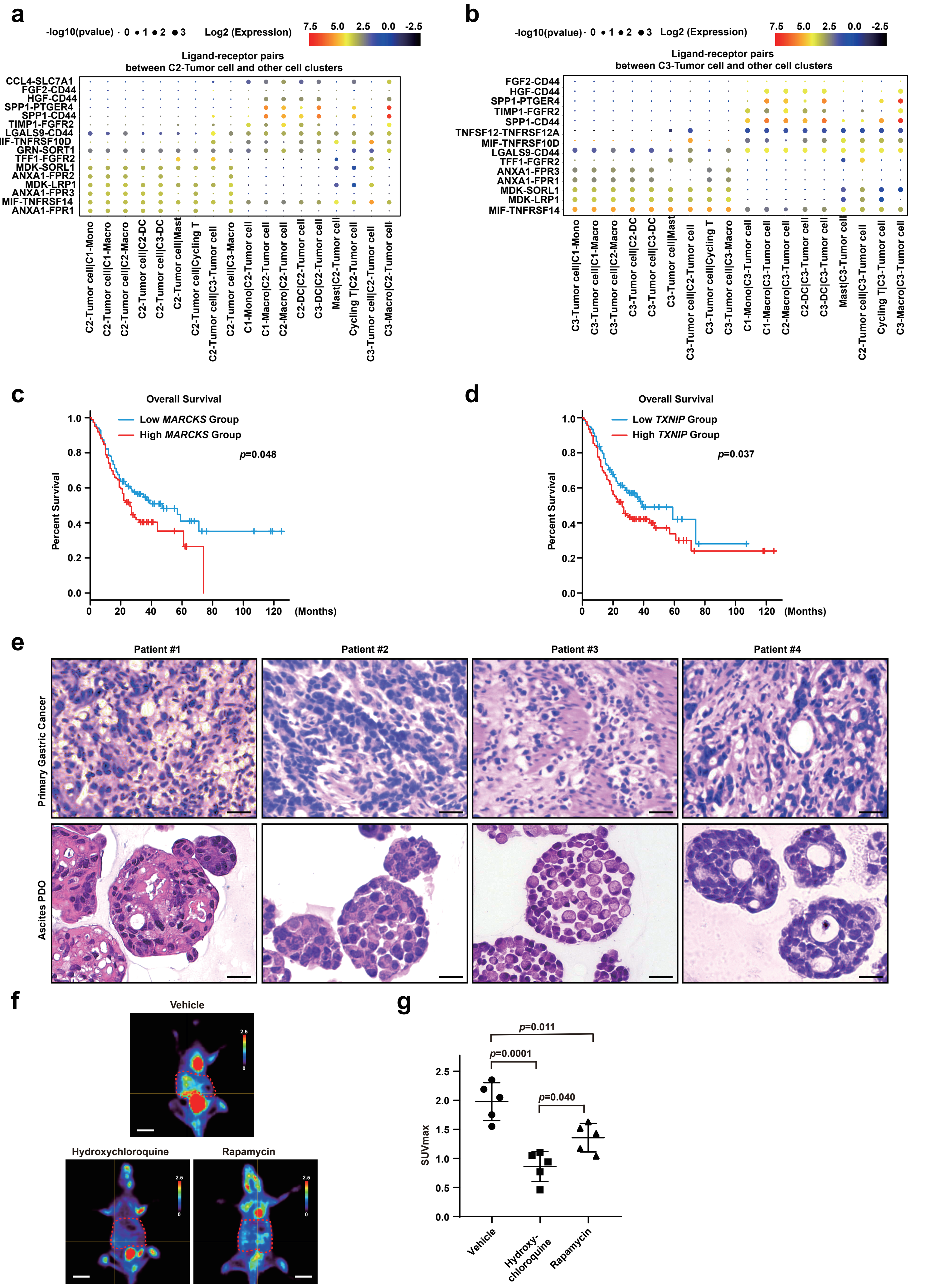
a
-log10(pvalue) · 0 • 1 • 2 • 3
Log2 (Expression)
7.5 5.0 2.5 0.0 -2.5
Ligand-receptor pairs
between C2-Tumor cell and other cell clusters
CCL4-SLC7A1
FGF2-CD44
HGF-CD44
SPP1-PTGER4
SPP1-CD44
TIMP1-FGFR2
LGALS9-CD44
MIF-TNFRSF10D
GRN-SORT1
TFF1-FGFR2
MDK-SORL1
ANXA1-FPR2
MDK-LRP1
ANXA1-FPR3
MIF-TNFRSF14
ANXA1-FPR1
C2-Tumor cell|C1-Mono
C2-Tumor cell|C1-Macro
C2-Tumor cell|C2-Macro
C2-Tumor cell|C2-DC
C2-Tumor cell|C3-DC
C2-Tumor cell|Mast
C2-Tumor cell|Cycling T
C2-Tumor cell|C3-Tumor cell
C2-Tumor cell|C3-Macro
C1-Mono|C2-Tumor cell
C1-Macro|C2-Tumor cell
C2-Macro|C2-Tumor cell
C2-DC|C2-Tumor cell
C3-DC|C2-Tumor cell
Mast|C2-Tumor cell
Cycling T|C2-Tumor cell
C3-Tumor cell|C2-Tumor cell
C3-Macro|C2-Tumor cell
b
-log10(pvalue) · 0 • 1 • 2 • 3
Log2 (Expression)
7.5 5.0 2.5 0.0 -2.5
Ligand-receptor pairs
between C3-Tumor cell and other cell clusters
FGF2-CD44
HGF-CD44
SPP1-PTGER4
TIMP1-FGFR2
SPP1-CD44
TNFSF12-TNFRSF12A
MIF-TNFRSF10D
LGALS9-CD44
TFF1-FGFR2
ANXA1-FPR3
ANXA1-FPR1
MDK-SORL1
MDK-LRP1
MIF-TNFRSF14
C3-Tumor cell|C1-Mono
C3-Tumor cell|C1-Macro
C3-Tumor cell|C2-Macro
C3-Tumor cell|C2-DC
C3-Tumor cell|C3-DC
C3-Tumor cell|Mast
C3-Tumor cell|C2-Tumor cell
C3-Tumor cell|Cycling T
C3-Tumor cell|C3-Macro
C1-Mono|C3-Tumor cell
C1-Macro|C3-Tumor cell
C2-Macro|C3-Tumor cell
C2-DC|C3-Tumor cell
C3-DC|C3-Tumor cell
Mast|C3-Tumor cell
C2-Tumor cell|C3-Tumor cell
Cycling T|C3-Tumor cell
C3-Macro|C3-Tumor cell
c
Overall Survival
Low MARCKS Group
High MARCKS Group
p=0.048
Percent Survival
1.0 0.8 0.6 0.4 0.2 0.0
0 20 40 60 80 100 120 (Months)
d
Overall Survival
Low TXNIP Group
High TXNIP Group
p=0.037
Percent Survival
1.0 0.8 0.6 0.4 0.2 0.0
0 20 40 60 80 100 120 (Months)
e
Patient #1
Patient #2
Patient #3
Patient #4
Primary Gastric Cancer
Ascites PDO
f
Vehicle
Hydroxychloroquine
Rapamycin
2.5
0
g
p=0.011
p=0.0001
p=0.040
SUVmax
2.5 2.0 1.5 1.0 0.5 0.0
Vehicle
Hydroxy-chloroquine
Rapamycin

Supplementary Fig. 7. Characteristics of tumor cell involved in paligenosis and gastric cancer patient-derived organoids

(a,b) Dotplot showing intercellular interaction between C2-Tumor cell (a) and C3-Tumor cell (b) and immune cell subtypes, based on ligand-receptor pairs by CellPhoneDB analysis. Dot size represents the significance of the interactions and dot color represents expression levels.

(c,d) Kaplan-Meier curves showing the overall survival of patients from The Cancer Genome Atlas Stomach Adenocarcinoma (TCGA-STAD) database with high (red line) or low (blue line) expression group of *MARCKS* (c) and *TXNIP* (d), respectively. *p* value is calculated by log-rank test.

(e) Hematoxylin-eosin staining for matched patient biopsies (top) and patient-derived organoid from ascites (bottom), showing that the organoids maintain the growth patterns and differentiation of the primary gastric cancer. Scale bars, 100 μm.

(f) 18F-FDG PET images of mice at the five weeks after intraperitoneal injection with PDOs. Scale bar, 2cm.

(g) Quantification of SUVmax in patient-derived organoid xenograft (PDOX) as in (f) (n = 5 mice in each group). Data are presented as mean values ± SEM (error bars); *p* values are calculated by one-way ANOVA with Tukey post hoc test.

Supplementary Table and Supplementary Table Legends

Supplementary Table 1. Summary of scRNA-seq patient sample information.

Patient ID	Age(years)	Sex	Group	Sample type	Pathologic AJCC stage	Therapeutic history
HA_7	42-66	Female	G0	Normal peritoneal lavage fluid	N/A*	Treatment-naïve
HA_12	42-66	Female	G0	Normal peritoneal lavage fluid	N/A*	Treatment-naïve
HA_42	42-66	Female	G0	Normal peritoneal lavage fluid	N/A*	Treatment-naïve
HA_43	42-66	Female	G0	Normal peritoneal lavage fluid	N/A*	Treatment-naïve
HA_11	38-63	Male	G1	Peritoneal lavage fluid	pT1bN0M0, IA	Treatment-naïve
HA_14	38-63	Male	G1	Peritoneal lavage fluid	pT1aN0M0, IA	Treatment-naïve
HA_16	38-63	Male	G1	Peritoneal lavage fluid	pT1aN0M0, IA	Treatment-naïve
HA_40	38-63	Female	G1	Peritoneal lavage fluid	pT1aN0M0, IA	Treatment-naïve
HA_1	52-84	Male	G2	Peritoneal lavage fluid	pT2N2M0, IIB	Treatment-naïve
HA_10	52-84	Male	G2	Peritoneal lavage fluid	pT3N1M0, IIB	Treatment-naïve
HA_20	52-84	Male	G2	Peritoneal lavage fluid	pT3N2M0, IIIA	Treatment-naïve
HA_21	52-84	Male	G2	Peritoneal lavage fluid	pT4aN1M0, IIIA	Treatment-naïve
HA_26	52-84	Male	G2	Peritoneal lavage fluid	pT3N0M0, IIA	Treatment-naïve
HA_37	52-84	Male	G2	Peritoneal lavage fluid	pT4aN2M0, IIIA	Treatment-naïve
HA_38	52-84	Female	G2	Peritoneal lavage fluid	pT3N3aM0, IIIB	Treatment-naïve
HA_39	52-84	Male	G2	Peritoneal lavage fluid	pT4aN2M0, IIIA	Treatment-naïve
HA_41	52-84	Male	G2	Peritoneal lavage fluid	pT2N0M0, IB	Treatment-naïve
HA_48	52-84	Male	G2	Peritoneal lavage fluid	pT3N1M0, IIB	Treatment-naïve
HA_2	36-81	Male	G3	Ascites	N/A*	Treatment-naïve
HA_3	36-81	Male	G3	Ascites	N/A*	Treatment-naïve
HA_6	36-81	Male	G3	Ascites	N/A*	Treatment-naïve
HA_9	36-81	Male	G3	Ascites	N/A*	Treatment-naïve
HA_18	36-81	Female	G3	Ascites	N/A*	Treatment-naïve
HA_27	36-81	Male	G3	Ascites	N/A*	Treatment-naïve
HA_28	36-81	Female	G3	Ascites	N/A*	Treatment-naïve
HA_30	36-81	Female	G3	Ascites	N/A*	Treatment-naïve
HA_31	36-81	Female	G3	Ascites	N/A*	Treatment-naïve
HA_32	36-81	Male	G3	Ascites	N/A*	Treatment-naïve
HA_35	36-81	Male	G3	Ascites	N/A*	Treatment-naïve
HA_36	36-81	Mele	G3	Ascites	N/A*	Treatment-naïve
HA_25	46-83	Female	G4	Ascites	N/A*	S-1+Paclitaxel
HA_29	46-83	Male	G4	Ascites	N/A*	S-1+Paclitaxel+Apatinib
HA_33	46-83	Female	G4	Ascites	N/A*	S-1+Atezolizumab
HA_34	46-83	Male	G4	Ascites	N/A*	S-1+Oxaliplatin+Toripalimab
HA_44	46-83	Female	G4	Ascites	N/A*	S-1+Oxaliplatin

N/A*: Not applicable due to benign hysteromyoma or gastric cancer peritoneal metastasis without surgery, age as a range for each patient within each group.

Supplementary Table 2. Summary of scRNA-seq transcriptomic profiles.

Sample	Group	Cell counts per sample	Median genes per cell	Median UMI counts per cell	Mitochondrial genes per cell (%)
HA_7	G0	4689	2010	5006	5.58
HA_12	G0	4746	3177	10412	4.25
HA_42	G0	4111	3913	14720	6.47
HA_43	G0	6035	3205	10927	6.50
HA_11	G1	2707	3028	9961	4.34
HA_14	G1	2689	4057	18129	6.41
HA_16	G1	5483	2390	6962	6.16
HA_40	G1	5889	3675	13036	6.92
HA_1	G2	5393	2321	6389	7.33
HA_10	G2	6099	2954	8847	7.02
HA_20	G2	5680	2030	6703.5	5.56
HA_21	G2	6423	1867	5096	6.08
HA_26	G2	5926	1996.5	5126.5	4.99
HA_37	G2	5699	2217	6060	5.63
HA_38	G2	5957	2368	6313	5.51
HA_39	G2	4456	2969	9866.5	7.08
HA_41	G2	6341	2832	8706	5.82
HA_48	G2	4179	3352	13019	6.18
HA_2	G3	8844	1731.5	5823.5	8.62
HA_3	G3	5018	2056	6740	5.04
HA_6	G3	5130	1931	6295	5.29
HA_9	G3	4796	2008	6045.5	4.51
HA_18	G3	5038	2430.5	7579.5	5.81
HA_27	G3	5320	2172	6054.5	5.08
HA_28	G3	6066	3890	15862.5	5.54
HA_30	G3	6470	2674	7387	6.26
HA_31	G3	6480	1603	4834.5	6.69
HA_32	G3	5933	2062	6945	4.88
HA_35	G3	3406	1989	5888	5.03
HA_36	G3	7147	3655	14717	8.78
HA_25	G4	12655	1790	5030	4.36
HA_29	G4	5786	1442.5	4004	6.60
HA_33	G4	3658	1972	5222	5.43
HA_34	G4	2701	1702	4173	4.23
HA_44	G4	5037	3141	10494	7.72

UMI: Unique Molecular Identifier

Supplementary Table 3. Summary of PDOs sample information.

Patient ID	Age	Gender	Sample type	AJCC stage
PDO#1	57-83	Male	Ascites	N/A*
PDO#2	57-83	Male	Ascites	T4N3bM1
PDO#3	57-83	Male	Ascites	N/A*
PDO#4	57-83	Female	Ascites	T3N3aM1

N/A*: Not applicable due to gastric cancer peritoneal metastasis without surgery; PDOs: patient-derived organoids.

Supplementary Table 4. Antibodies application and dilution

Antibodies	Company	Application	Dilution
Mouse Anti-TXNIP (JY2)	Novus (NBP1-54578)	IF	1:50
Mouse Anti-β-Tubulin	Cell Signaling Technology (2146S)	IF	1:200
Rabbit Anti-MARCKS (D88D11)	Cell Signaling Technology (5607S)	IF	1:100
Rabbit Anti-LC3B	Cell Signaling Technology (2775S)	IF	1:50
Rabbit Anti-SOX9	Merck (AB5535)	IF	1:100
Mouse Anti-SOX9	Abcam (ab76997)	IF	1:100
Rabbit Anti-REDD1	Proteintech (10638-1-AP)	IF	1:100
Rabbit Anti-ATF3	Sigma (HPA001562)	IF	1:200
Rabbit Anti-PS6 Ribosomal Protein (Ser235/236)	Cell Signaling Technology (4858S)	IF	1:250
Rabbit Anti-Ki67 (SP6)	Thermo Fisher Scientific (MA5-14520)	IF	1:100
Donkey anti-Mouse IgG (H+L) Highly Cross-Adsorbed Secondary Antibody, Alexa Fluor Plus 594	Invitrogen (A-21203)	IF	1:500
Donkey anti-Mouse IgG (H+L) Highly Cross-Adsorbed Secondary Antibody, Alexa Fluor Plus 488	Invitrogen (A-21202)	IF	1:200
Donkey anti-Rabbit IgG (H+L) Highly Cross-Adsorbed Secondary Antibody, Alexa Fluor Plus 594	Invitrogen (A-21207)	IF	1:500
Donkey anti-Rabbit IgG (H+L) Highly Cross-Adsorbed Secondary Antibody, Alexa Fluor Plus 488	Invitrogen (A-21206)	IF	1:200
Anti-human CD45-eFluor	eBioscience (69-0459-42)	FACS	5 μL/test
Anti-human CD3-FITC	eBioscience (11-0037-42)	FACS	5 μL/test
Anti-human CD56-FITC	eBioscience (11-0566-42)	FACS	5 μL/test
Anti-human CD19-FITC	eBioscience (11-0199-42)	FACS	5 μL/test
Anti-human CD1c-APC	BioLegend (331524)	FACS	5 μL/test
Anti-human CD163-PE	eBioscience (12-1639-42)	FACS	5 μL/test
Anti-human CD14-BV421	BD Biosciences (563743)	FACS	5 μL/test
7-AAD Viability Staining Solution	eBioscience (00-6993-50)	FACS	5 μL/test

Abbreviation, IF: Immunofluorescence; FACS: Fluorescence-activated cell sorting.
