# Supplementary material for: Single-cell sequencing of ascites fluid illustrates heterogeneity and therapy-induced evolution during gastric cancer peritoneal metastasis
Source: Nat Commun. 2023 Feb 14;14:822. doi: 10.1038/s41467-023-36310-9 (PMC9929081; doi:10.1038/s41467-023-36310-9)
Supplement: Supplementary file 2 — Reporting Summary [file 41467_2023_36310_MOESM2_ESM.pdf]

nature portfolio

Corresponding author(s): Zhen-Ning Wang

Last updated by author(s): Jan 16, 2023

Reporting Summary

Nature Portfolio wishes to improve the reproducibility of the work that we publish. This form provides structure for consistency and transparency in reporting. For further information on Nature Portfolio policies, see our Editorial Policies and the Editorial Policy Checklist.

Statistics

For all statistical analyses, confirm that the following items are present in the figure legend, table legend, main text, or Methods section.

n/a	Confirmed	
☐	☒	The exact sample size (*n*) for each experimental group/condition, given as a discrete number and unit of measurement
☐	☒	A statement on whether measurements were taken from distinct samples or whether the same sample was measured repeatedly
☐	☒	The statistical test(s) used AND whether they are one- or two-sided<br>*Only common tests should be described solely by name; describe more complex techniques in the Methods section.*
☐	☒	A description of all covariates tested
☐	☒	A description of any assumptions or corrections, such as tests of normality and adjustment for multiple comparisons
☐	☒	A full description of the statistical parameters including central tendency (e.g. means) or other basic estimates (e.g. regression coefficient) AND variation (e.g. standard deviation) or associated estimates of uncertainty (e.g. confidence intervals)
☐	☒	For null hypothesis testing, the test statistic (e.g. *F*, *t*, *r*) with confidence intervals, effect sizes, degrees of freedom and *P* value noted<br>*Give P values as exact values whenever suitable.*
☒	☐	For Bayesian analysis, information on the choice of priors and Markov chain Monte Carlo settings
☐	☒	For hierarchical and complex designs, identification of the appropriate level for tests and full reporting of outcomes
☐	☒	Estimates of effect sizes (e.g. Cohen's *d*, Pearson's *r*), indicating how they were calculated

Our web collection on statistics for biologists contains articles on many of the points above.

Software and code

Policy information about availability of computer code

Data collection	All single-cell RNA sequencing libraries were constructed in Key Laboratory of Precision Diagnosis and Treatment of Gastrointestinal Tumors of Ministry of Education of China Medical University following the manufacturer's instructions of the 10X Genomics Chromium single cell 3' platform, and the constructed libraries were sequenced on Illumina Novaseq6000 sequencer. Raw scRNA-seq data were collected using Cell Ranger Software (version 4.0.0, 10X Genomics). ELISA data were collected using Tecan i-control Software (version 1.11, TECAN). FACS data were collected using BD FACS Diva Software (version 8.0.2, BD). All PDOs sizes were measured in Image J (v1.52a, NIH).
Data analysis	1) Cell Ranger (v4.0.0; https://support.10xgenomics.com/single-cell-gene-expression/software/pipelines/latest/what-is-cell-ranger) and STAR (v2.5.1b; https://github.com/alexdobin/STAR) were used for demultiplexing, barcode processing, read alignment, gene counting, and generation of gene-barcode expression matrix.<br>2) DoubletFinder (v2.0.1; https://github.com/chris-mcginnis-ucsf/DoubletFinder) was used to estimate and remove potential doublets.<br>3) Harmony (v1.0; https://github.com/immunogenomics/harmony) was used to integrate multiple scRNA-seq dataset and correct batch effect.<br>4) Seurat (v3.2.3; https://github.com/satijalab/seurat) was used to analyze single-cell RNA-seq data.<br>5) singleR (v1.0.1; https://github.com/dviraran/SingleR) was used for cell annotation.<br>6) inferCNV (v1.2.1; https://github.com/broadinstitute/inferCNV) was used to compute chromosomal copy number variations (CNV).<br>7) Monocle2 (v2.4.0; https://github.com/cole-trapnell-lab/monocle-release) was used to infer cell lineage developmental trajectory.<br>8) GSVA (v1.30.0; http://www.bioconductor.org/packages/release/bioc/html/GSVA.html) was used to estimate metabolism pathway activity.

9) CellPhoneDB (v2.0.0; https://www.cellphonedb.org/) was used to explore cell-cell interactions.
10) SCENIC (v0.10.3; https://github.com/aertslab/SCENIC) was used to estimate transcription factor activity.
11)All analyses were performed on the following softwares: R (v4.0.5; https://www.r-project.org/), Python (v3.9.0; https://www.python.org/), and GraphPad Prism (v8.0.1; https://www.graphpad.com).

For manuscripts utilizing custom algorithms or software that are central to the research but not yet described in published literature, software must be made available to editors and reviewers. We strongly encourage code deposition in a community repository (e.g. GitHub). See the Nature Portfolio guidelines for submitting code & software for further information.

Data

Policy information about availability of data

All manuscripts must include a data availability statement. This statement should provide the following information, where applicable:

- Accession codes, unique identifiers, or web links for publicly available datasets
- A description of any restrictions on data availability
- For clinical datasets or third party data, please ensure that the statement adheres to our policy

The single-cell RNA sequencing data involves phenotype and genotype data for human subjects. Notably, our data is only limited to health-related scientific research and is not permitted for other commercial use. In addition, this single-cell RNA sequencing data is also involved in our ongoing project. Therefore, the single-cell RNA sequencing data generated in this study have been deposited in the Genome Sequence Archive for Human (GSA-Human) database under accession code HRA002712 (https://ngdc.cncb.ac.cn/gsa-human/browse/HRA002712), with appropriate access control. The single-cell RNA sequencing data included in this study are available upon reasonable request by contact with the corresponding author (Zhen-Ning Wang, josieon826@sina.cn) and will be responded to request within two weeks with access granted and duration of data use based on the applicant's research needs. The processed single-cell RNA sequencing data are available at Source Data file. The detailed data for Figures and Supplementary Figures are summarized in Source Data file. Source Data are provided with this paper.

Human research participants

Policy information about studies involving human research participants and Sex and Gender in Research.

Reporting on sex and gender	Sex information is provided in Supplementary Table 1.
Population characteristics	The detailed patient ID, age, group allocation, sample type, and AJCC stage of patients are provided in Supplementary Table 1. A total of 35 patients were included in this study, and all patients were adults (age range: 36-84). Of these, 4 donors were benign hysteromyoma patients; 4 donors were early gastric cancer patients; 10 donors were advanced gastric cancer patients; 12 donors were untreated patients with diagnosed gastric cancer peritoneal metastasis; and 5 donors were patients with diagnosed gastric cancer peritoneal metastasis following therapy.
Recruitment	We recruited the patients who were eligible for pathological diagnosis in the Affiliated Hospital of China Medical University, the Sheng Jing Hospital of China Medical University, the Liaoning Cancer Hospital & Institute, and the People’s Hospital of Liaoning Province following the ethical guidelines. Seventeen samples of malignant ascites fluid were collected from patients pathologically diagnosed with gastric cancer and 18 peritoneal lavage fluid samples were collected from gastric cancer patients without peritoneal metastasis or from patients pathologically diagnosed with benign hysteromyoma . Of these collected samples, four control peritoneal lavage fluid samples were taken from four benign hysteromyoma patients (negative controls, G0 Group); peritoneal lavage fluid from four early gastric cancer patients (G1 Group); peritoneal lavage fluid from 10 advanced gastric cancer patients (G2 Group); ascites from 12 untreated gastric cancer patients with diagnosed peritoneal metastasis (G3 Group) and ascites from five gastric cancer patients with diagnosed peritoneal metastasis following systemic therapy (G4 Group). There is no potential self-selection bias.
Ethics oversight	This study was performed following the ethical guidelines of the Declaration of Helsinki and was approved by the Research Ethics Committee of China Medical University (Shenyang, China). Informed consent was obtained from all enrolled patients before collection of samples and clinical information.

Note that full information on the approval of the study protocol must also be provided in the manuscript.

Field-specific reporting

Please select the one below that is the best fit for your research. If you are not sure, read the appropriate sections before making your selection.

[x] Life sciences [ ] Behavioural & social sciences [ ] Ecological, evolutionary & environmental sciences

For a reference copy of the document with all sections, see nature.com/documents/nr-reporting-summary-flat.pdf

Life sciences study design

All studies must disclose on these points even when the disclosure is negative.

Sample size	No sample size calculation was performed. We enrolled 35 patients, with a total of 191,987 high-quality single cells for further downstream analysis; thus the sample size is sufficient for single-cell analysis. The detailed information is: scRNA-seq transcriptomic profiles of cells from the peritoneal ecosystem were from 35 patients, including normal peritoneal lavage fluid from four benign hysteromyoma patients (negative

	controls, G0 group), peritoneal lavage fluid from four early gastric cancer (GC) patients (G1 group); peritoneal lavage fluid from 10 advanced GC patients (G2 group); ascites from 12 untreated patients with diagnosed gastric cancer peritoneal metastasis (GCPM, G3 group); and ascites from five patients with diagnosed GCPM following therapy (G4 group). Additional four ascites samples were collected to culture patient-derived ascites organoids for experimental validations. After quality control and cell filtering, a total of 191,987 high-quality cells were retained for further downstream analysis.
Data exclusions	Low-quality cells were filtered following these criteria: (1): cells with less than 200 genes; (2) cells with less than 800 UMI counts or ranked in the top 1% of UMI counts; (3) cells with more than 20% mitochondrial gene count. Genes detected in less than three cells were also excluded from downstream analyses. Subsequently, the "DoubletFinder" R package was used to predict and remove potential doublets.
Replication	The protocols of sample collection and single-cell suspension preparation were tested in more than two patients and the verified protocols were consistently used across the different samples. Single-cell suspension preparation, cell concentration and cell viability were simultaneously performed in triplicates and the sample was used for further scRNA-seq only when the results of the triplicates are similar. Single-cell data analyses, including dimensionality reduction, unsupervised cell clustering, differential expressed gene analysis, cell annotation, trajectory inference analysis, survival analysis, and visualization were performed three times, independently. Validation experiments based on patient ascites-derived organoids were done more than three replicates. All replications were successful and the results were reproducible. The detailed information was showed in corresponding figure and table legends.
Randomization	Random assignment is not applicable for human samples with different pathological diagnosis, due to tumor pathological staging can not been designed as randomization. In validation experiments, organoids and mice were randomly allocated into different inhibitors treatment.
Blinding	Researchers, who conducted experiments on sample collection, Single-cell RNA library preparation and sequencing, did not participate in data collection and data analysis. Researchers, who conducted data collection and data analysis, did not participate in experiments on sample collection, Single-cell RNA library preparation and sequencing. For animal experiments, the group information were marked with numbers. Researchers who conducted data collection and analysis were blinded to the group information. Regardless of the group assignment, the blinding exclusion of low-quality cells was performed based on corresponding criteria before single-cell data analyses. Moreover, all dimensionality reduction, unsupervised cell clustering, differential expressed gene analysis, cell annotation, trajectory inference analysis, survival analysis, and visualization were performed using unsupervised or blinded approaches.

Reporting for specific materials, systems and methods

We require information from authors about some types of materials, experimental systems and methods used in many studies. Here, indicate whether each material, system or method listed is relevant to your study. If you are not sure if a list item applies to your research, read the appropriate section before selecting a response.

Materials & experimental systems

n/a	Involved in the study	
☐	☒	Antibodies
☒	☐	Eukaryotic cell lines
☒	☐	Palaeontology and archaeology
☐	☒	Animals and other organisms
☒	☐	Clinical data
☒	☐	Dual use research of concern

Methods

n/a	Involved in the study	
☒	☐	ChIP-seq
☐	☒	Flow cytometry
☒	☐	MRI-based neuroimaging

Antibodies

Antibodies used

Antibodies used for immunofluorescence staining:
Mouse Anti-TXNIP (JY2), Novus (NBP1-54578), used at 1:50 dilution
Mouse Anti-β-Tublin, Cell Signaling Technology (2146S), used at 1:200 dilution
Rabbit Anti-MARCKS (D88D11), Cell Signaling Technology (5607S), used at 1:100
Rabbit Anti-LC3B, Cell Signaling Technology (2775S), used at 1:50 dilution
Rabbit Anti-SOX9, Merck (AB5535), used at 1:100 dilution
Mouse Anti-SOX9, Abcam (ab76997), used at 1:100 dilution
Rabbit Anti-REDD1, Proteintech (10638-1-AP), 1:100 dilution
Rabbit Anti-ATF3, Sigma (HPA001562), used at 1:200 dilution
Rabbit Anti-PS6 Ribosomal Protein (Ser235/236), Cell Signaling Technology (4858S), used at 1:250 dilution
Rabbit Anti-Ki67 (SP6), Thermo Fisher Scientific (MA5-14520), used at 1:100 dilution
Donkey anti-Mouse IgG (H+L) Highly Cross-Adsorbed Secondary Antibody, Alexa Fluor Plus 594, Invitrogen (A-21203), used at 1:500 dilution
Donkey anti-Mouse IgG (H+L) Highly Cross-Adsorbed Secondary Antibody, Alexa Fluor Plus 488, Invitrogen (A-21202), used at 1:200 dilution
Donkey anti-Rabbit IgG (H+L) Highly Cross-Adsorbed Secondary Antibody, Alexa Fluor Plus 594, Invitrogen (A-21207), used at 1:500 dilution
Donkey anti-Rabbit IgG (H+L) Highly Cross-Adsorbed Secondary Antibody, Alexa Fluor Plus 488, Invitrogen (A-21206), used at 1:200 dilution

Antibodies used for FACS:

	Anti-human CD45-eFluor (Cat# 69-0459-42, eBioscience™), used at 5 µL/test<br>Anti-human CD3-FITC (Cat# 11-0037-42, eBioscience™), used at 5 µL/test<br>Anti-human CD56-FITC (Cat# 11-0566-42, eBioscience™), used at 5 µL/test<br>Anti-human CD19-FITC (Cat# 11-0199-42, eBioscience™), used at 5 µL/test<br>Anti-human CD1c-APC (Cat# 331524, BioLegend), used at 5 µL/test<br>Anti-human CD163-PE (Cat# 12-1639-42, eBioscience™), used at 5 µL/test<br>Anti-human CD14-BV421(Cat# 563743, DB Biosciences) , used at 5 µL/test<br>7-AAD Viability Staining Solution (Cat# 00-6993-50, eBioscience™), used at 5 µL/test
Validation	All of the antibodies used in this study were validated for IF and FACS use in human specimens by both the manufacturers and our pre-experiment. The detailed antibody information and antibody validation procedures were described on the following respective manufacturers' websites: https://www.cellsignal.com, https://www.abcam.com, https://www.sigmaaldrich.com, https://www.thermofisher.cn/cn/zh/home.html, and https://www.ptgcn.com, https://www.novusbio.com, https://www.biolegend.com and https://www.bdbiosciences.com.

Animals and other research organisms

Policy information about studies involving animals; ARRIVE guidelines recommended for reporting animal research, and Sex and Gender in Research

Laboratory animals	NOD/SCID mice were purchased from Beijing Vital River Laboratory Animal Technology (Beijing, China).
Wild animals	Wild animal were not involved in the study.
Reporting on sex	NOD/SCID mice involved in this study were all female.
Field-collected samples	No field-collected samples are involved in the study.
Ethics oversight	All animal experiments were performed in accordance with the National Institutes of Health Guide for Care and Use of Laboratory Animals, the ARRIVE guidelines, and the institutional ethical guidelines (China Medical University Animal Studies Committees).

Note that full information on the approval of the study protocol must also be provided in the manuscript.

Flow Cytometry

Plots

Confirm that:

- [x] The axis labels state the marker and fluorochrome used (e.g. CD4-FITC).
- [x] The axis scales are clearly visible. Include numbers along axes only for bottom left plot of group (a 'group' is an analysis of identical markers).
- [x] All plots are contour plots with outliers or pseudocolor plots.
- [x] A numerical value for number of cells or percentage (with statistics) is provided.

Methodology

Sample preparation	Ascites fluids were immediately transported to our laboratory on ice following drainage. Liquid samples were aliquoted into 50-ml centrifuged tubes through 70-um cell strainers (Cat# 352350, BD FALCON) and centrifuged at 300g for 5 min at 4 °C. After supernatant removal, the remaining cell pellet was resuspended in red blood cell lysis buffer (Cat# R1010, Solarbio) and incubated on ice for 5 min to remove red blood cell contamination. Phosphate-buffered saline (PBS; Cat# SH30256.01, HyClone) was used to quench the red blood cell lysis buffer and then the cell suspension was centrifuged at 300g for 5 min at 4 °C to pellet the cells. The above process of red blood cell lysis was repeated until no red blood cells were visible. The resulting cell pellet was washed twice with PBS then resuspended in sorting buffer (PBS containing 2% fetal bovine serum [FBS; Cat# DT-100-S, DearyTech]). The cells for FACS were stained with Fluorophore-conjugated antibodies containing CD45-eFluor (Cat# 69-0459-42, eBioscience™), CD3-FITC (Cat# 11-0037-42, eBioscience™), CD56-FITC (Cat# 11-0566-42, eBioscience™), CD19-FITC (Cat# 11-0199-42, eBioscience™), CD1c-APC (Cat# 331524, BioLegend), CD163-PE (Cat# 12-1639-42, eBioscience™), CD14-BV421(Cat# 563743, DB Biosciences) on ice for 30 minutes. After washing twice with FACS buffer, the cells were stained using 7-AAD Viability Staining Solution (Cat# 00-6993-50, eBioscience™) on ice 5 minutes.
Instrument	FACSAriaIII (BD Bioscience)
Software	BD FACS Diva Software (version 8.0.2, BD)
Cell population abundance	In gastric cancer ascites: CD45+ live cell was ~90% of single cell population; CD1C+and Lineage- (cDC2) was ~2% of the CD45+ live population; CD14+and CD163+was ~50% of the cDC2 population.<br>In gastric cancer peritoneal lavage fluid: CD45+ live cell was ~95% of single cell population; CD1C+and Lineage- (cDC2) was ~5% of the CD45+ live population; CD14+and CD163+was ~20% of the cDC2 population.

Gating strategy	SSC-A and FSC-A were used to gate single cells; CD45 and 7AAD were used to gate CD45+ live cells; CD1C and Lineage (CD3, CD56 and CD19) were used to gate cDC2 cells; CD14 and CD163 were used to gate monocyte-like dendritic cells.

☒ Tick this box to confirm that a figure exemplifying the gating strategy is provided in the Supplementary Information.
