# Supplementary material for: Single-cell sequencing of ascites fluid illustrates heterogeneity and therapy-induced evolution during gastric cancer peritoneal metastasis
Source: Nat Commun. 2023 Feb 14;14:822. doi: 10.1038/s41467-023-36310-9 (PMC9929081; doi:10.1038/s41467-023-36310-9)
Supplement: Supplementary file 3 — Description of Additional Supplementary Files [file 41467_2023_36310_MOESM3_ESM.docx]

**Description of Additional Supplementary Files**

File Name: Supplementary Data 1

Description: The detailed gene signatures for cell function.
